# Critical role for cholesterol in Lassa fever virus entry identified by a novel small molecule inhibitor targeting the viral receptor LAMP1

**DOI:** 10.1371/journal.ppat.1007322

**Published:** 2018-09-28

**Authors:** May Kwang-Mei Wang, Tao Ren, Hu Liu, Sun-Young Lim, Kyungae Lee, Anna Honko, Huanying Zhou, Julie Dyall, Lisa Hensley, Ashley K. Gartin, James M. Cunningham

**Affiliations:** 1 Division of Hematology, Department of Medicine, Brigham and Women’s Hospital, Boston, Massachusetts, United States of America; 2 Department of Microbiology and Immunobiology, Harvard Medical School, Boston, Massachusetts, United States of America; 3 United States Army Medical Research institute of Infectious Disease, Fort Detrick, Maryland, United States of America; 4 NIAID/NIH Integrated Research Facility, Fort Detrick, Maryland, United States of America; Thomas Jefferson University, UNITED STATES

## Abstract

Lassa fever virus (LASV) is endemic in West Africa and causes severe hemorrhagic fever and sensorineural hearing loss. We identified a small molecule inhibitor of LASV and used it to analyze the mechanism of entry. Using a photo-reactive analog that retains antiviral activity as a probe, we identified the inhibitor target as lysosome-associated membrane protein 1 (LAMP1), a host factor that binds to the LASV glycoprotein (GP) during infection. We found that LAMP1 binding to LASV GP is cholesterol-dependent, and that the inhibitor blocks infection by competing with cholesterol in LAMP1. Mutational analysis of a docking-based model identified a putative inhibitor binding site in the cholesterol-binding pocket within the LAMP1 domain that binds GP. These findings identify a critical role for cholesterol in LASV entry and a potential target for therapeutic intervention.

## Author summary

Lassa fever virus (LASV) is endemic in West Africa and can cause fatal infection. Currently, there is no vaccine or effective post-exposure treatment. Here we identify 3.3, a small molecule that inhibits LASV infection by targeting lysosome-associated membrane protein 1 (LAMP1), which binds to the LASV glycoprotein (GP) and promotes virus membrane fusion and infection. Our analysis reveals that cholesterol is an important cofactor for LAMP1 binding to LASV GP and that 3.3 inhibits infection by displacing cholesterol from LAMP1. Thus, 3.3 is a candidate for further development as a LASV inhibitor.

## Introduction

Lassa fever virus (LASV) is a highly pathogenic enveloped RNA virus that is endemic in western Africa [1, 2]. In early studies of LASV infection, the surface membrane protein α-dystroglycan (α-DG) was identified as a glycoprotein (GP) attachment factor and a key determinant of tissue tropism. LASV particles bind to α-DG and are transported to late endosomes and lysosomes (LE/LY) where exposure to pH <5.0 triggers GP-mediated membrane fusion and infection [3–5]. More recent studies have indicated that interactions of LASV with the host during infection are significantly more complex. First, it has been found that LASV tropism is not restricted to cells that express α-DG. Candidate alternative attachment factors have been identified and include the well-characterized TIM and TAM family proteins that are recognized attachment factors for other families of viruses, including filoviruses [6–8]. In addition, a genetic screen for host factors mediating LASV entry identified the LE/LY membrane protein LAMP1 [9]. Detailed studies of the role of LAMP1 revealed that in the acidic LE/LY, LASV GP dissociates from α-DG and binds to LAMP1 [9–11], which markedly enhances virus membrane fusion and infection [12, 13]. Studies in cultured cells show that binding of GP to LAMP1 is not strictly required for acid pH to trigger LASV infection [13]. However, virus propagation was not detected in LAMP1 knockout mice injected with LASV [9]. Herein, we report the identification of a small molecule that inhibits LASV infection by targeting LAMP1 and interfering with a critical interaction with cholesterol that promotes LAMP1 binding to LASV GP.

## Results

### The small molecule 3.3 is a specific inhibitor of Lassa fever virus

**Fig 1:**
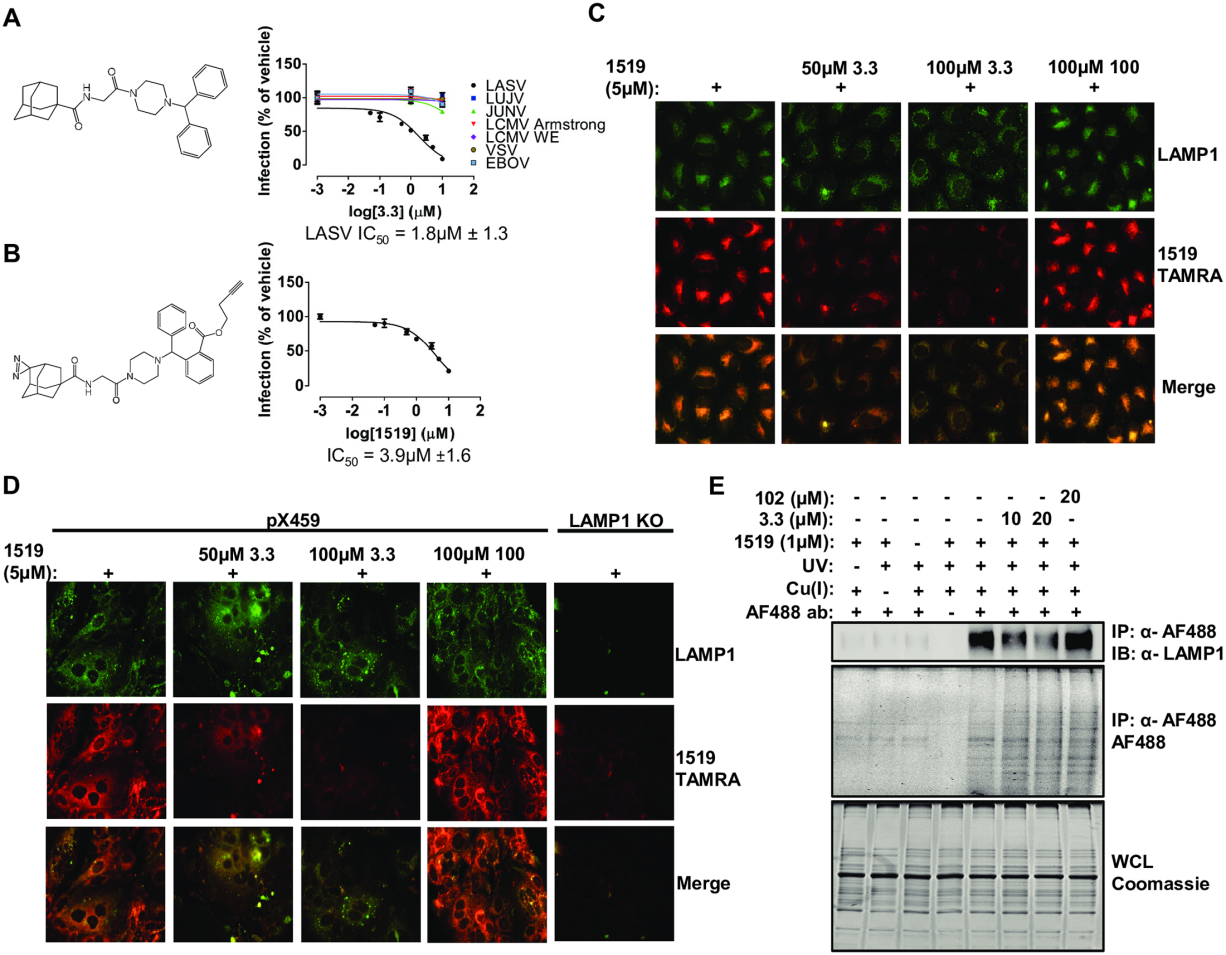
3.3 inhibits LASV GP-mediated infection and cross-links to the LASV receptor, LAMP1, in cells. (A) Structure of 3.3 (left) and inhibition of transduction by MLV pseudotyped with the indicated viral glycoproteins (right). Vero cells were incubated with the indicated concentrations of 3.3 for 1h before challenge with MLV encoding GFP and pseudotyped with the indicated viral glycoproteins. Virus transduction is reported as % of GFP-positive cells relative to cells exposed to DMSO vehicle alone. Data are mean ± SD (*n* = 3). IC_50_ is the concentration of inhibitor required to reduce transduction by 50%. LUJV: Lujo virus; JUNV: Junin virus; LCMV: Lymphocytic choriomeningitis virus; VSV: Vesicular stomatitis virus; EBOV: Ebola virus. (B) Structure of 1519 (left). 1519 contains a diazirine for target cross-linking and a terminal alkyne for Cu^+^-catalyzed click-based analysis and inhibits transduction by MLV pseudotyped with LASV GP (right). Vero cells were incubated with the indicated concentrations of 1519 for 1h before challenge with MLV pseudotyped with LASV GP and encoding GFP. Virus transduction is reported as % of GFP-positive cells relative to cells exposed to DMSO vehicle alone. Data are mean ± SD (*n* = 3). Data was collected in the same experiment as Fig 1A. (C) 1519 co-localizes with LAMP1+ compartments in Vero cells and cross-links in a manner which is competed by the addition of a molar excess of 3.3 but not the inactive 3.3 derivative, 100. Vero cells were co-incubated with 1519 and the indicated competitor compound for 1h at 37°C before UV irradiation followed by click chemistry with TAMRA azide. LAMP1 was visualized by immunofluorescence using an anti-LAMP1 antibody. (D) 1519 co-localizes with LAMP1+ compartments in Huh7.5 cells and cross-links in a manner which is competed by the addition of a molar excess of 3.3 but not the inactive 3.3 derivative, 100. 1519 cross-linking in Huh7.5 LAMP1 knockout (KO) cells is diminished. Huh7.5 pX459 or LAMP1 KO cells were co-incubated with the indicated compounds for 1h at 37°C before UV irradiation followed by click chemistry with TAMRA azide. LAMP1 was visualized by immunofluorescence using an anti-LAMP1 antibody. (E) 1519 cross-links to endogenous LAMP1 in a manner which is competed by the addition of a molar excess of 3.3 but not the inactive 3.3 derivative, 102. Vero cells were incubated with the indicated compounds for 1h at 37°C before UV irradiation. Cells were harvested, lysed and used for click chemistry to attach an Alexa Fluor 488 (AF 488) azide and then subjected to immunoprecipitation using an anti-AF 488 antibody. AF 488 labeled LAMP1 was detected using an anti-LAMP1 antibody. Total AF 488 labeled proteins were detected via in-gel fluorescence. Input protein was detected by Coomassie staining.

A hit from a screen to discover small molecule inhibitors of virus infection identified the adamantyl diphenyl piperazine 3.3 as an inhibitor of transduction of Vero cells by murine leukemia virus (MLV) particles pseudotyped with LASV GP (IC_50_ = 1.8μM ± 1.3), but not of transduction by MLV particles pseudotyped with GPs from the related Old World arenaviruses lymphocytic choriomeningitis virus (LCMV) Armstrong, LCMV WE, LuJo virus (LUJV) or with Junin virus (JUNV), Ebola virus (EBOV) or vesicular stomatitis virus (VSV) (Fig 1A). In addition, the infection of Vero cells by LASV engineered to encode eGFP (rLASV-eGFP) was inhibited more than 90% by 3.3 (S1C Fig).

### 3.3 targets the viral receptor LAMP1 in cells

To identify the target of 3.3, the derivative 1519 was synthesized. 1519 is a less potent inhibitor of LASV GP-mediated infection than 3.3 (IC_50_ = 3.9μM ±1.6), but contains a photoreactive diazirine moiety suitable for formation of covalent adducts with target proteins and an alkyne moiety for coupling 1519 adducts to azides using bio-orthogonal Cu^+^-catalyzed click chemistry (Fig 1B). Vero cells incubated with 1519 were UV-irradiated, washed and fixed, and 1519 adducts were “click”-coupled to the fluorophore TAMRA-azide. Imaging of these cells showed that the TAMRA signal strongly co-localized with the LE/LY membrane protein LAMP1 in cytoplasmic vesicles and that labeling by 1519 was attenuated by co-incubating cells with a 10- or 20-fold excess of 3.3, but not by a 20-fold excess of the inactive 3.3 derivative, 100, indicating that 1519 cross-links the 3.3 target (Fig 1C and S1 Fig). Since LASV infection is strongly hepatotropic [14], we also tested human hepatocyte-derived Huh7.5 cells and observed robust 1519 labeling that was specifically inhibited by co-incubation with 3.3 and by deletion of the LAMP1 gene (Fig 1D, S1 and S2 Figs). To determine if LAMP1 is a direct target of 1519, Vero cells were labeled with 1519 and click-conjugated to Alexa Fluor 488 (AF 488)-azide. Lysates of these cells were prepared, and labeled proteins were immunoprecipitated using an antibody specific for AF 488 and analyzed for LAMP1 by immunoblot. The results demonstrate that 1519 forms a covalent adduct with LAMP1 and that formation of the 1519-LAMP1 adduct is specifically inhibited by a molar excess of 3.3, but not by a molar excess of the inactive 3.3 derivative 102 (Fig 1E and S1 Fig).

### 3.3 inhibits LAMP1 binding to LASV GP

**Fig 2:**
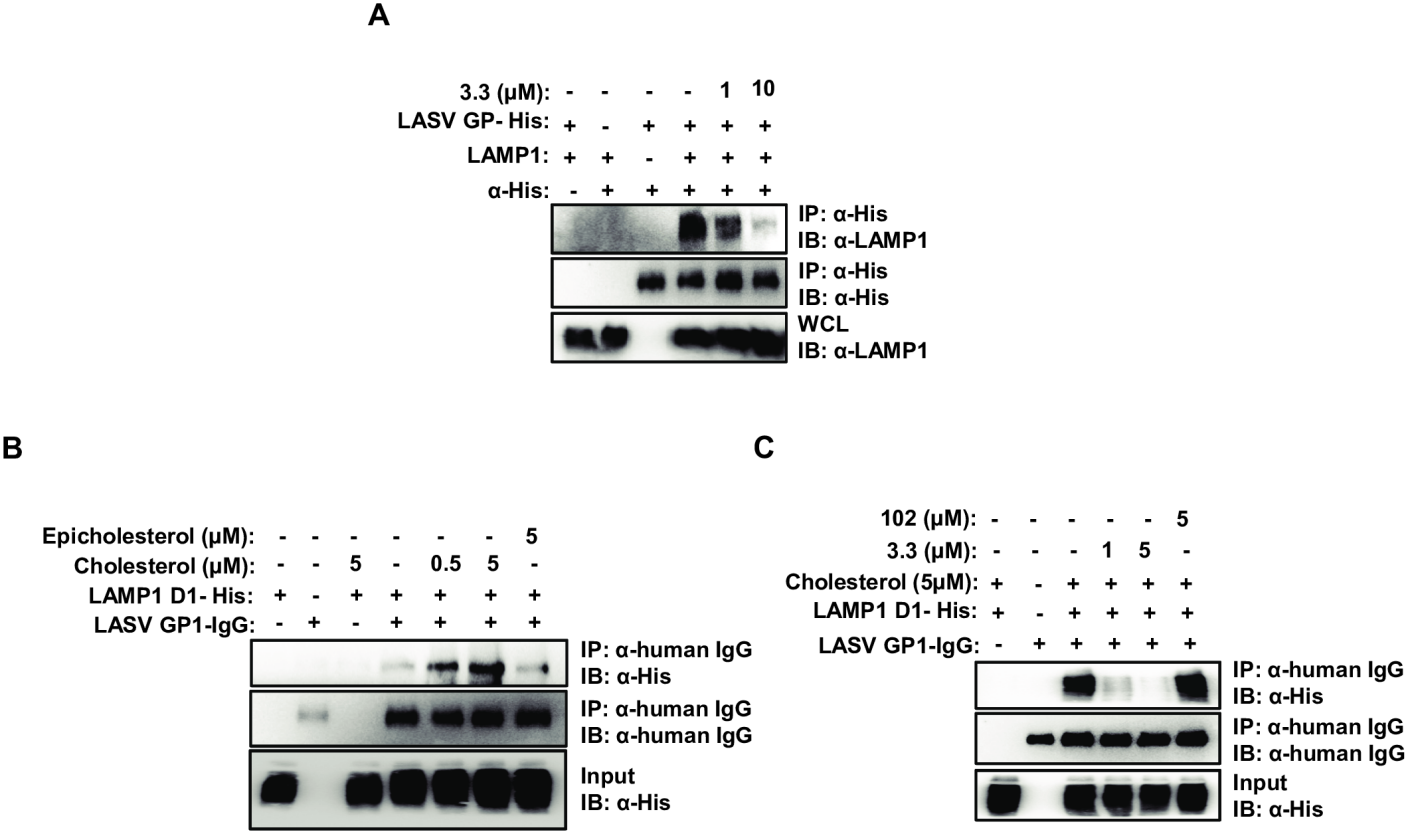
Binding of LASV GP to LAMP1 is dependent on cholesterol and is inhibited by 3.3. (A) 3.3 blocks the interaction of LASV GP with endogenous LAMP1. 293T LAMP1 KO cells expressing LASV GP-His and 293T pX459 cells were incubated with the indicated concentrations of 3.3 for 1h at 37°C before lysis. Lysates were mixed and subjected to immunoprecipitation with an anti-His antibody. The presence of LASV GP bound LAMP1 was detected by immunoblot with an anti-LAMP1 antibody. (B) LASV GP1-IgG binds to LAMP1 D1 in a cholesterol-dependent manner. Purified LAMP1 D1-His was incubated with the indicated concentrations of cholesterol or epicholesterol for 1h at 37°C before the addition of purified LASV GP1-IgG. Samples were subjected to immunoprecipitation against human IgG and bound LAMP1 D1 was detected by immunoblot with an anti-His antibody. (C) 3.3 inhibits the cholesterol-dependent binding of LASV GP1-IgG to LAMP1 D1. Purified LAMP1 D1-His was co-incubated with 5μM cholesterol and the indicated concentrations of 3.3 or 102 for 1h at 37°C before the addition of purified LASV GP1-IgG. Samples were subjected to immunoprecipitation against human IgG and bound LAMP1 D1 was detected by immunoblot with an anti-His antibody.

To examine the effect of 3.3 on LASV GP binding to LAMP1, lysates from LAMP1 knockout (KO) 293T cells (S2 Fig) expressing LASV GP-His and from 293T pX459 cells treated with 3.3 were prepared and mixed at pH 5.5. LASV GP was immunoprecipitated and LAMP1 binding to LASV GP was analyzed by immunoblot. The results confirm that LASV GP binds to LAMP1 and show that binding is inhibited by 3.3 (Fig 2A). Taken together with the results of the cross-linking experiments using 1519, these findings strongly suggest that 3.3 inhibits LASV infection by targeting LAMP1 and interfering with LAMP1 binding to LASV GP.

### GP binding to LAMP1 D1 is cholesterol-dependent and sensitive to 3.3 inhibition

Next, we investigated the molecular basis for LAMP1 binding to LASV GP. The 3.3 target LAMP1 is a type I membrane protein in which the ectodomain consists of two related “LAMP” domains [15–17]. A recent study demonstrated that LASV GP-mediated entry depends on the presence of the membrane distal LAMP1 domain D1 [9]. To investigate the effect of 3.3 on the interaction between D1 and LASV GP, a LASV GP1-IgG fusion protein was purified and validated as a ligand for endogenous LAMP1 from cells (S3 Fig). In the initial studies, immunoprecipitation of purified, soluble D1 by LASV GP1-IgG was weak (Fig 2B). A recent study reported that the LAMP domains in LAMP1 (and also LAMP2) reversibly bind to cholesterol [15]. To determine if cholesterol is required for LASV GP binding, purified D1 binding to LASV GP1-IgG was examined after incubation of D1 with cholesterol. We found that the addition of cholesterol increased binding of D1 to LASV GP1-IgG in a dose-dependent manner. By contrast, incubation of D1 with the 3’-OH cholesterol enantiomer epicholesterol, which does not bind to LAMP [15], did not enhance D1 binding to LASV GP1-IgG (Fig 2B). Importantly, the cholesterol-dependent binding of D1 to LASV GP1-IgG was inhibited by the presence of 3.3, but not by the inactive 3.3 derivative, 102 (Fig 2C and S1 Fig). Taken together, these findings confirm that D1 binds to LASV GP1 and demonstrate that binding is cholesterol-dependent and sensitive to inhibition by 3.3.

### Cholesterol and 3.3 stabilize the tertiary structure of LAMP1

**Fig 3:**
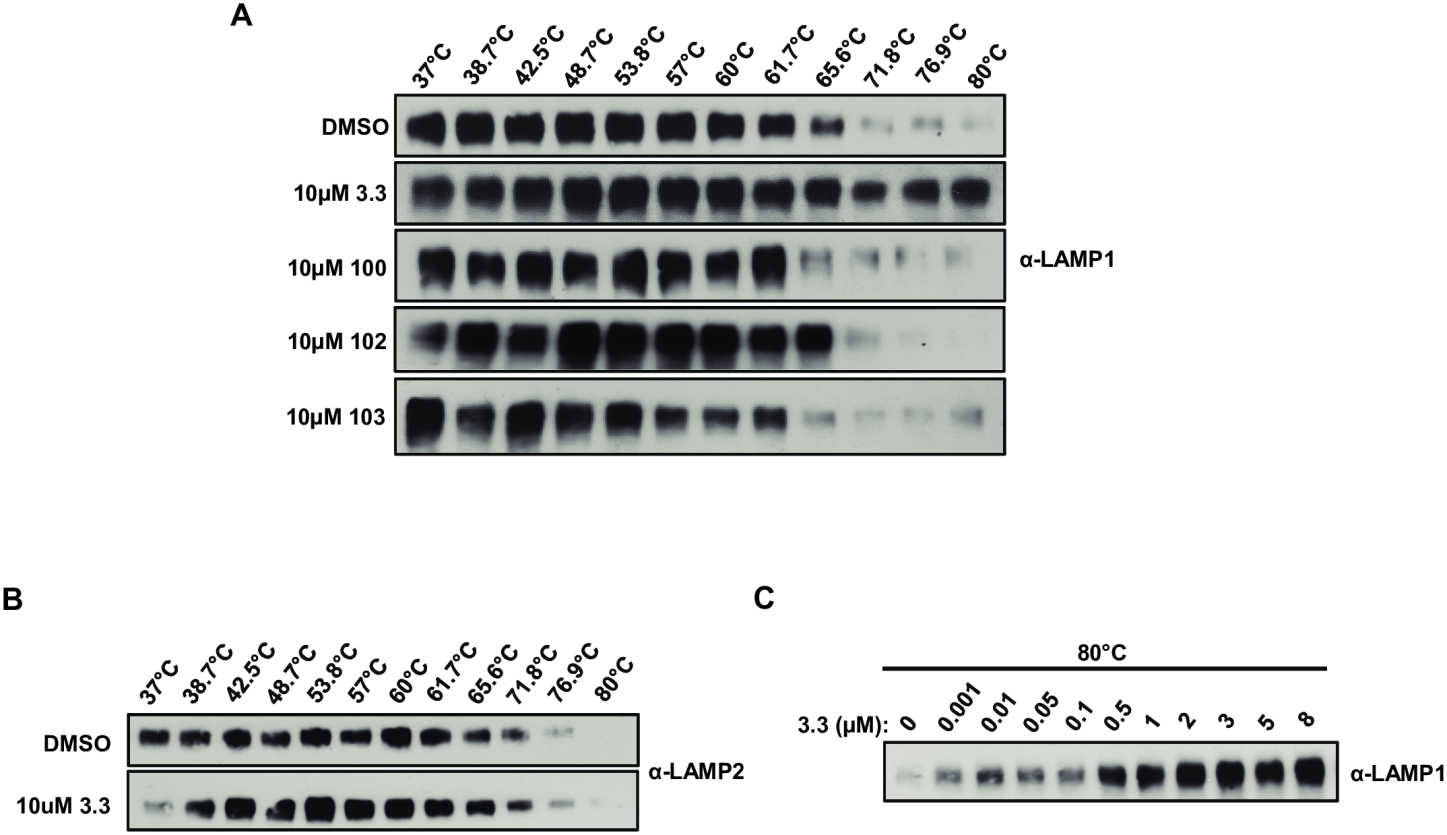
3.3 protects LAMP1, but not LAMP2, from thermal denaturation in cells. (A) 3.3 but not the inactive derivatives, 100, 102 and 103, raises the T_m_ of endogenous LAMP1. 293T cells were incubated with 10μM of the indicated compounds or DMSO for 1h at 37°C. Cells were harvested and heated to the indicated temperatures prior to lysis. Lysed samples were centrifuged and the supernatants were analyzed by immunoblot with an anti-LAMP1 antibody. (B) 3.3 does not alter the thermal denaturation profile of endogenous LAMP2. 293T cells were incubated with 10μM 3.3 or DMSO for 1h at 37°C. Cells were harvested and heated to the indicated temperatures prior to lysis. Lysed samples were centrifuged and the supernatants were analyzed by immunoblot with an anti-LAMP2 antibody. (C) 3.3 dose-dependently protects endogenous LAMP1 from thermal denaturation at 80°C. 293T cells were incubated with the indicated concentrations of 3.3 or DMSO for 1h at 37°C. Cells were harvested and heated to 80°C prior to lysis. Lysed samples were centrifuged and the supernatants were analyzed by immunoblot with an anti-LAMP1 antibody.

We pursued the mechanism of 3.3 inhibition of LASV GP binding to D1. Previous work indicated that cholesterol binding to LAMP1 is reversible and that the cholesterol binding site is located within a hydrophobic pocket in the center of the pyramid-shaped LAMP domain [15–17]. We used thermal denaturation profiling to analyze the effect of cholesterol binding on LAMP1. Purified D1 was heated to a specific temperature between 60°C and 80°C for 3 minutes. After cooling, denatured, aggregated D1 was pelleted by centrifugation, and the supernatant was analyzed by immunoblot. We found that D1 is nearly completely denatured at 74.7°C (S4A Fig). Pre-incubation of D1 with cholesterol, but not epicholesterol, protected D1 from thermal denaturation at 80°C (S4B Fig). Notably, 3.3 also protected D1 from thermal denaturation at 80°C (S4C Fig). Thus, both cholesterol and 3.3 stabilize the tertiary structure of D1. The thermal denaturation assay was used to analyze the effect of 3.3 on LAMP1 in cells [18]. The temperature profiles for denaturation of endogenous LAMP1 was similar to that of purified D1 (Fig 3A). Treatment of cells with 3.3 protected LAMP1, but not LAMP2, from denaturation up to 80°C (Fig 3A and 3B). The dose-response relationship of 3.3 on the thermal stability of LAMP1 at 80°C closely correlated with the antiviral activity of 3.3 (Fig 3C) and accordingly, treatment of cells with the inactive 3.3 derivatives 100, 102 or 103 had no discernable effects on the thermal inactivation profile of LAMP1 (Fig 3A and S1 Fig). These findings demonstrate that the effect of 3.3 on purified D1 *in vitro* is representative of its effect on LAMP1 in cells.

### 3.3 is a competitive inhibitor of cholesterol binding to LAMP1

**Fig 4:**
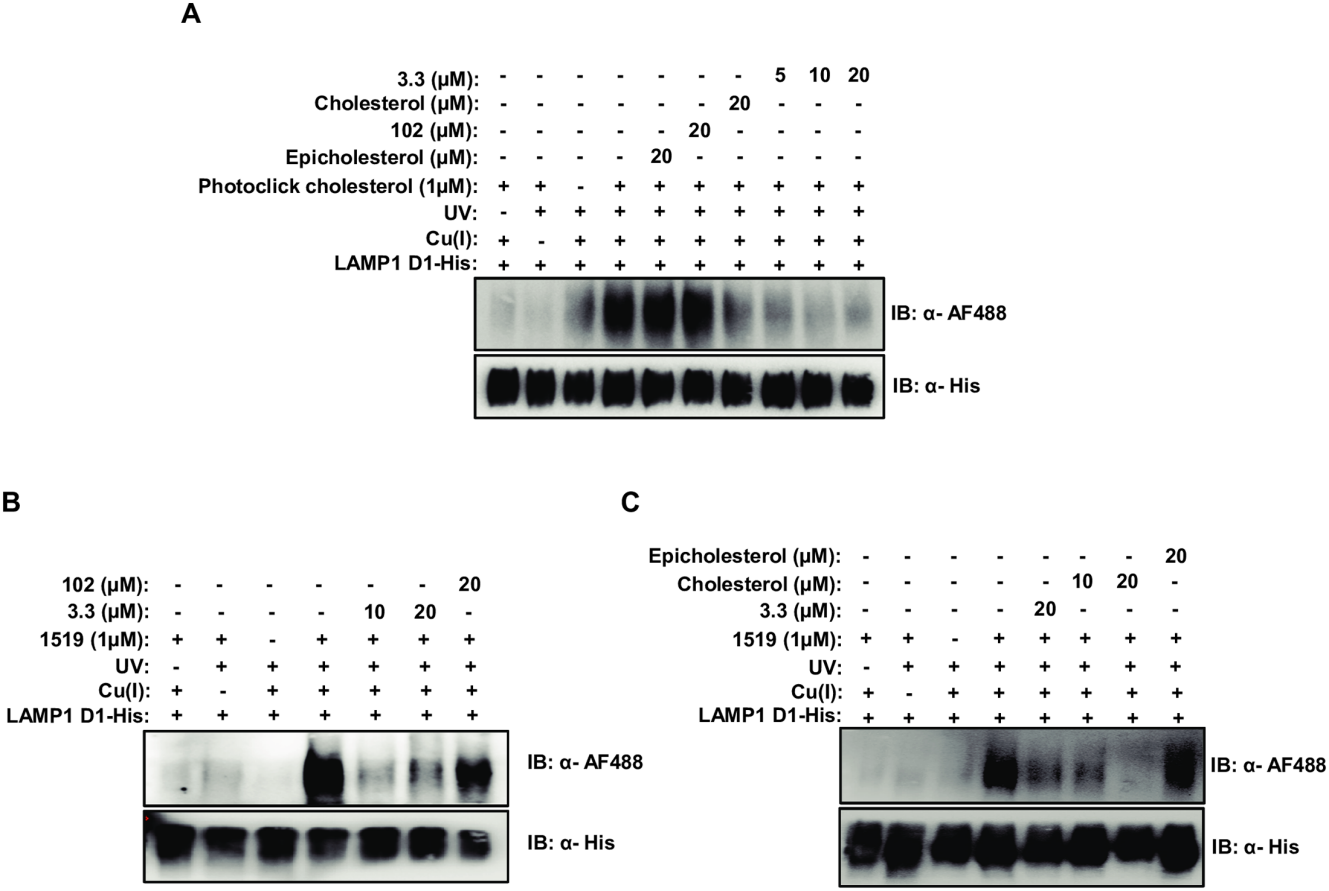
Cross-linking of 1519 to LAMP1 D1 is inhibited by cholesterol and vice versa. (A) A photoreactive cholesterol analog (photoclick cholesterol) cross-linking to purified LAMP1 D1 is competed by a molar excess of cholesterol or 3.3 but not by a molar excess of epicholesterol or 102. Purified LAMP1 D1-His was incubated with the indicated compounds for 1h at 37°C prior to UV irradiation and click chemistry with an AF 488 azide. Labeled protein was detected by immunoblot with an anti-AF 488 antibody. Input LAMP1 D1 was detected using an anti-His antibody. (B) 1519 cross-links to purified LAMP1 D1 in a manner which is competed by a molar excess of 3.3 but not the inactive derivative, 102. Purified LAMP1 D1-His was incubated with the indicated compounds for 1h at 37°C prior to UV irradiation and click chemistry with an AF 488 azide. Labeled protein was detected by immunoblot with an anti-AF 488 antibody. Input LAMP1 D1 was detected using an anti-His antibody. (C) 1519 cross-links to purified LAMP1 D1 in a manner which is competed by a molar excess of 3.3 and cholesterol but not epicholesterol. Purified LAMP1 D1-His was incubated with the indicated concentrations of 1519 and 3.3, cholesterol or epicholesterol for 1h at 37°C prior to UV irradiation and click chemistry with an AF 488 azide. Labeled protein was detected by immunoblot with an anti-AF 488 antibody and input LAMP1 D1 was detected using an anti-His antibody.

The findings that the antiviral activity of 3.3 is cholesterol-dependent and that the stabilizing effect of 3.3 on the thermal denaturation profile of D1 is similar to that of cholesterol suggested that 3.3 might also bind in the central pocket. We employed 1519 and a photo-reactive cholesterol analog with a diazirine and terminal alkyne [19] as probes to address this question. Upon photo-activation, the cholesterol analog formed a covalent adduct with D1 that was inhibited by the presence of either cholesterol or 3.3, but not by epicholesterol or the inactive 3.3 derivative 102 (Fig 4A). Similarly, cross-linking of 1519 to D1 was inhibited by 3.3 and by cholesterol, but not by 102 or by epicholesterol (Fig 4B and 4C). Thus, 3.3 is a competitive inhibitor of cholesterol binding to D1 and vice versa.

### Model of 3.3 binding to LAMP1

**Fig 5:**
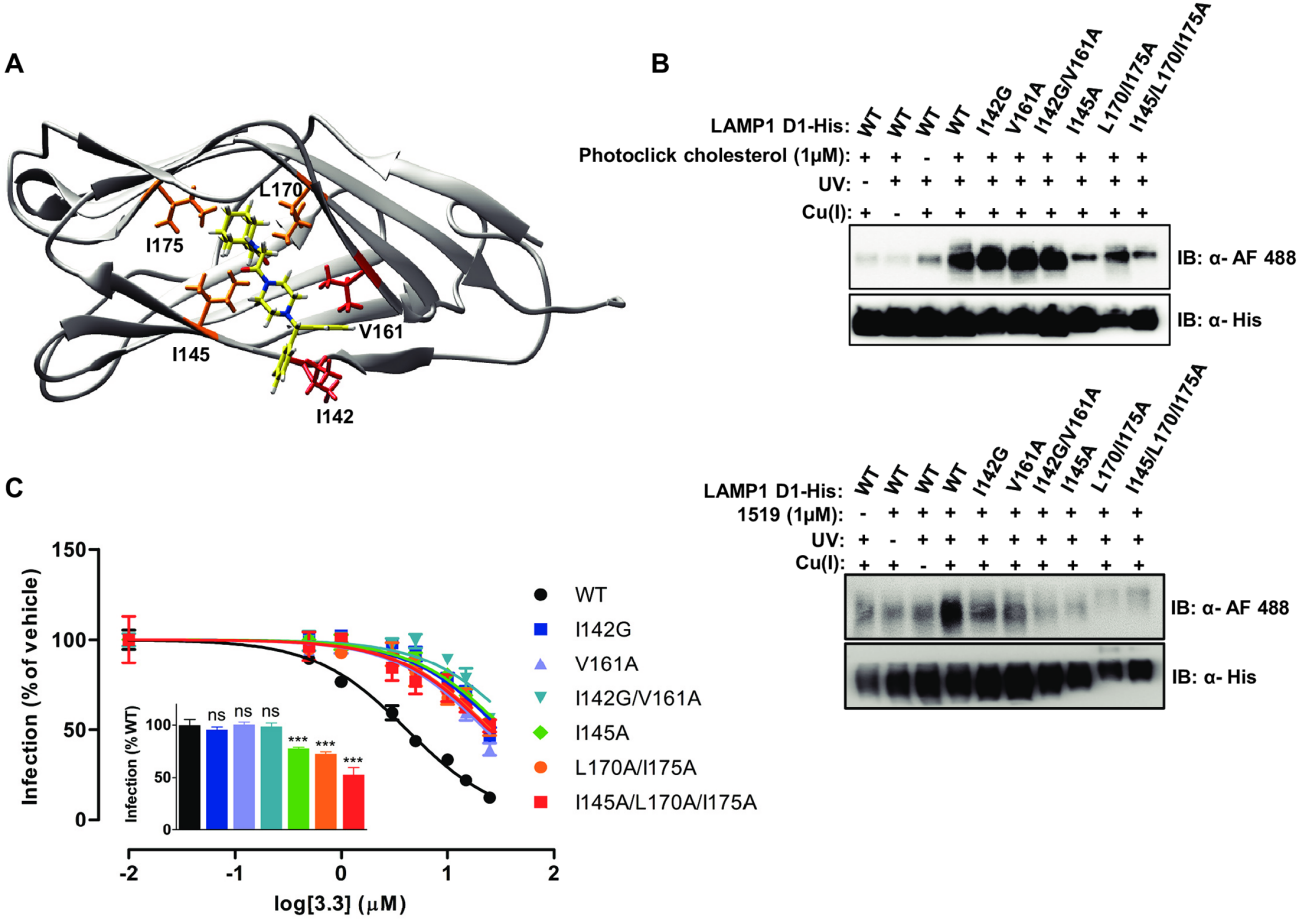
3.3 binding to the hydrophobic central pocket of LAMP1 D1. (A) Docking of 3.3 (yellow) in the predicted structure of LAMP1 D1. Residues within the hydrophobic pocket predicted to contact 3.3 are colored orange. Residues on the surface at the edge of the pocket predicted to contact the diphenyl group of 3.3 are colored red. (B) Mutational analysis of predicted 3.3 contacts with LAMP1 D1 on cross-linking by photoclick cholesterol and by 1519. Purified LAMP1 D1 containing the indicated mutations were incubated with photoclick cholesterol (top) or 1519 (bottom) for 1h at 37°C prior to UV irradiation and click chemistry with AF 488 azide. Labeled protein was detected by immunoblot with an anti-AF 488 antibody. Input LAMP1 D1 was detected using an anti-His antibody. (C) Mutational analysis of predicted 3.3 contacts with LAMP1 D1 on transduction by MLV LASV GP and 3.3 inhibition. 293T LAMP1 KO cells transfected with the indicated LAMP1 mutants were incubated with the indicated concentrations of 3.3 for 1h before challenge with MLV pseudotyped with LASV GP and encoding GFP. Virus transduction is reported as % of GFP-positive cells relative to cells exposed to DMSO vehicle alone. Inset shows the MLV LASV GP transduction efficiency relative to that on cells transfected with wild-type (WT) LAMP1. Data are mean ± SD (*n* = 3). The IC_50_ of the WT curve differs significantly from all other curves (*p* < 0.001). Inset: ns (not significant); *** (*p* < 0.001). Statistical analyses were performed using one-way ANOVA with Tukey post-hoc analysis.

We employed a docking program to identify a candidate 3.3 binding site in D1. In the docking-based model, the 3.3 adamantane and dipeptide moieties are buried in the central pocket of D1 in proximity to the side chains of residues I145, L170 and I175, and the 3.3 diphenyl rings are adjacent to the side chains of residues I142 and V161 that reside at the edge of the pocket on the surface of D1 (Fig 5A and S5 Fig). We tested this model by measuring the effects of substitutions for these residues on purified D1 and on LAMP1 in cells. We found that the substitutions I145A, L170A/I175A and I145A/L170A/I175A located within the pocket decreased the efficiency of cross-linking by both 1519 and photoclick cholesterol to purified D1 (Fig 5B). The substitutions I142G, V161A and I142G/V161A at the edge of the pocket also reduced cross-linking by 1519, but not by photoclick cholesterol (Fig 5B). In line with this, substitutions within the pocket reduced both LAMP1 LASV receptor activity and sensitivity to 3.3 inhibition of infection whereas substitutions at the edge of the pocket also reduced the sensitivity of LAMP1 to 3.3, but had no effect on receptor activity (Fig 5C). These findings demonstrate that the role of LAMP1 in LASV entry is sensitive to changes in the central pocket of D1 that interfere with cholesterol binding and that 3.3 binds in the central pocket of D1 in a way that is also dependent on contacts with residues I142 and V161.

## Discussion

In this report, we demonstrate that the function of LAMP1 as a host factor for LASV infection is strongly dependent on bound cholesterol. This conclusion is based on studies of the mechanism of action of 3.3, a newly identified small molecule inhibitor of LASV infection that targets LAMP1. Using a photo-reactive analog of 3.3 that forms a covalent adduct with LAMP1, we show that 3.3 is a competitive inhibitor of cholesterol binding to D1, the LAMP1 domain that binds to LASV GP during infection. Our findings support a model in which the binding sites for 3.3 and for cholesterol reside within the hydrophobic pocket in the center of the prism-shaped D1. This model is consistent with the proposed location of the cholesterol-binding site in the closely-related LAMP2 [15] and also with the observation that both cholesterol and 3.3 increase the thermal stability of LAMP1 D1. Structural studies of LASV GP bound to D1 are needed to determine if cholesterol is part of the GP1 binding site or alternatively, if cholesterol, but not 3.3, is an allosteric regulator of the receptor-active conformation of D1.

Previous studies of the role of LAMP1 in infection indicate that acidification of the LE/LY reduces the affinity of LASV for α-DG, enhances binding to LAMP1, and promotes virus membrane fusion [9–13]. In this scheme, LAMP1 binding may function in concert with acid pH to activate the intrinsic membrane fusion activity of LASV GP analogous to the cooperative roles of receptor binding and endosomal acidification in alpharetrovirus infection [20, 21]. LAMP1 may also promote infection by positioning LASV particles in proximity to the target membrane. Relevant to the studies of LAMP1 receptor function reported here, acidification of LE/LY is also the signal for release of cholesterol-rich low density lipoprotein (LDL) particles transported by the LDL receptor [22]. LDL is the major source of exogenous cholesterol for the cell, and the release and transport of LDL-derived cholesterol across the LE/LY membrane is a multi-step process requiring de-esterification, extraction from specialized membrane vesicles by NPC2, and transport across the limiting membrane by NPC1. Based on the recent findings showing that LAMP1 and LAMP2 bind cholesterol reversibly and that LAMP2 resides in a complex with NPC1, it was proposed that LAMP proteins may also play a role in uptake of LDL cholesterol [15]. Further studies are needed to examine this hypothesis with a focus on possible consequences for LASV LAMP1 receptor function, LASV tropism and pathogenesis. An additional related issue that awaits further investigation is whether cholesterol has a role in the function of α-DG, TIM or TAM proteins as LASV attachment factors.

3.3 was identified in an analysis of the structure-activity relationship (SAR) of 3.0 (S1 Fig) [23, 24], a hit in a phenotypic screen of a small molecule library for specific inhibitors of EBOV infection. Analogous to the effect of 3.3 on LAMP1, 3.0 specifically targets the EBOV receptor NPC1 and interferes with its function in binding to EBOV GP and in cholesterol transport [23, 24]. The similarities in the structure and function of 3.0 and 3.3 suggest that the 3.0 target may be a cholesterol-binding site in NPC1 that is similar to the hydrophobic pocket in LAMP1 D1 that binds to 3.3. The model of 3.3 binding to D1 based on our studies predicts that the adamantane piperazine dipeptide that is present in both 3.0 and 3.3 resides in the central hydrophobic pocket that binds to cholesterol and the unique diphenyl moiety in 3.3 contacts residues I142 and V161 that are located just outside of the pocket. If this model is correct, it suggests that it may be possible to synthesize additional analogs of 3.0 and 3.3 that contain the adamantane piperazine dipeptide core and selectively target cholesterol binding sites in other proteins. We propose that screening these analogs may identify inhibitors of other enveloped viruses, such as alpha-, bunya- and hepadnaviruses for which a role for cholesterol in viral entry has been reported [25–29]. In addition, 3.3 may also be useful in examining the proposed role of LAMP1 in trafficking of LDL cholesterol.

LASV infection is associated with significant mortality and survivors have a high incidence of sensorineural hearing loss. 3.3 has several properties that suggest it has potential as an antiviral drug: (1) 3.3 is not cytotoxic to cultured cells, and knockout mice lacking the 3.3 target LAMP1 develop and function normally [30]; (2) 3.3 is membrane permeable and has a pK_a_ >8.0 and thus is expected to be selectively concentrated at the site of its target LAMP1 in the LE/LY, as we observed in cell-based labeling of LAMP1 with 1519 (Fig 1C); (3) 3.3 is specific for LAMP1 in that it did not target the closely related LE/LY protein LAMP2, as assessed by the thermal shift assay (Fig 3B), or NPC1, as indicated by the absence of anti-EBOV activity (Fig 1A). Recently, we completed a SAR campaign to improve the pharmacokinetic properties of the closely related Ebola-specific inhibitor 3.0 [31] that provides a framework for the optimization of 3.3 to identify derivatives for testing *in viv*o.

## Materials and methods

### Cell lines

Vero, HEK293T and Huh7.5 cell lines were obtained from ATCC (Manassas, VA) and maintained in DMEM (Invitrogen) supplemented with 10% FBS (Gemini Bio-Products) and 0.292mg/mL L-glutamine (Invitrogen) and grown at 37°C in 5% CO_2_. LAMP1 knockout and control pX459 cell lines (293T and Huh7.5) were generated by CRISPR. LAMP1 CRISPR gRNAs targeting exon 2 (5’-GAAGTTGGCCATTATGCACG-3’) and exon 6 (5’- GCTTGCTTGTCTTATGCAGA-3’) were designed using CHOPCHOP (http://chopchop.cbu.uib.no/) and cloned into a pSpCas9(BB)-2A-Puro (PX459) (a gift from Feng Zhang (Addgene plasmid #48139)) [32]. 293T and Huh7.5 cells were transfected with the CRISPR plasmids and selected with 2μg/mL puromycin. Knockout was confirmed by sequencing using primers amplifying the target site for exon 2 and exon 6 (5’-TGCACTCCAAGAGCAGCTGTCA-3’ and 5’-GACCCAGGGCACAAAAAGATGT-3’) and by immunoblot using a rabbit monoclonal anti-LAMP1 antibody (Cell Signaling Technologies) and a mouse monoclonal anti-tubulin antibody (Santa Cruz Biotechnology).

### Expression plasmids

Full length LASV (Josiah) GP (obtained from Gary Nabel, National Vaccine Center) was modified by adding a C-terminal 3xHA-6xHis tag and cloned into the pCAGGS expression vector. LASV GP1 (aa 1–255) fused to the N-terminus of human IgG was cloned into pCAGGS. Full length human LAMP1 and LAMP2 (obtained from Addgene) with a GSTGSTGSTGA linker, and LAMP1 D1 (aa 1–227) were fused at the C-terminus to a 3xFLAG-6xHis tag and cloned into the expression vector pCAGGS.

### Production of pseudotyped virions

Moloney murine leukemia virus (MLV) particles encoding green fluorescent protein (GFP) and pseudotyped with the indicated GPs were produced as previously described [33].

### Production and purification of LAMP1 D1 and Lassa GP1-IgG recombinant proteins

Recombinant proteins were produced and purified as previously described [33] with the exception that LASV GP1-IgG and LCMV GP1-IgG were purified on protein A/G agarose (Santa Cruz Biotechnology) and eluted using IgG elution buffer (Thermo Fisher).

### Infection assays with pseudotyped viruses

Infection assays were performed as previously described [33]. For experiments involving expression of mutant LAMP1 proteins, 293T LAMP1 KO cells plated on poly-L-lysine coated plates were transfected with the indicated LAMP1 proteins. One day after transfection, cells were treated with the indicated concentrations of 3.3 in a final concentration of 1% DMSO for 1h at 37°C before addition of LASV GP-pseudotyped MLV. IC_50_ values were calculated by non-linear regression curve-fitting using GraphPad Prism. Statistical analyses were performed using GraphPad Prism.

### rLASV-eGFP inhibition assay

Vero CCL81 cells were treated with 8 doses of 2-fold dilutions of 3.3 in media containing 1% DMSO. One hour later, cells were infected with a MOI = 0.3 of rLASV-eGFP. 48h post infection, plates were fixed in formalin and fluorescence was read on a Tecan plate reader (Infinite M1000) and on the Operetta High Content Imaging System. Compound was present throughout the entire experiment. Cytotoxicity was determined using the Cell Titer-Glo Luminescent Cell Viability Assay (Promega) after incubating cells with compound at the indicated concentrations for 48h. All procedures using infectious rLASV-eGFP were performed under biosafety level-4 (BSL-4) conditions.

### 1519 in-cell cross-linking/immunofluorescence

Stock solutions of LASV inhibitor 3.3 and 3.3 analogs 3.0, 100 and 1519 were diluted in media to the indicated concentrations (final DMSO 1.1%). Vero cells or Huh7.5 cells seeded on chambered coverglass (Nunc Lab-Tek II) were incubated with the compounds for 1h at 37°C in the dark. Cells were irradiated for 7min at 365nm followed by 3min at 302nm. Cells were then fixed with formalin and click chemistry was performed using 100μM TBTA, 2mM CuSO_4_, 1mM TCEP and 25μM TAMRA azide (Thermo Fisher) in 50mM Tris pH 8 by incubating at room temperature for 1h in the dark. Following click chemistry, cells were washed with PBS and permeabilized with 0.2% Triton and blocked with 1% BSA. LAMP1 was visualized using a mouse monoclonal LAMP1 antibody (Santa Cruz Biotechnology) followed by a goat anti-mouse Alexa Fluor 488 secondary antibody (Thermo Fisher). Cells were imaged on a Nikon Eclipse TE2000E.

### Cross-linking of photoreactive probes to LAMP1 FL and D1

#### 1519 cross-linking to LAMP1 FL

Vero cells were incubated with the indicated concentrations of 1519 and competitor compound in media (DMSO 1%) for 1h at 37°C in the dark. Cells were then irradiated for 7min at 365nm followed by 3min at 302nm. Cells were collected and lysed in lysis buffer (50mM HEPES pH 7.4, 150mM NaCl, 1% NP40) with protease inhibitor. Cell debris was pelleted by centrifugation at 4°C for 20min at 13,000RPM. SDS was added to the supernatants to a final concentration of 1% and the resulting mixture was subjected to click chemistry for 1h at room temperature to conjugate Alexa Fluor 488 azide (Thermo Fisher) to the 1519 alkyne (100μM TBTA, 2mM CuSO_4_, 1mM TCEP, 25μM Alexa Fluor 488 azide). Proteins were precipitated 5 times with cold acetone and the pellet was solubilized in 1% SDS lysis buffer. Prior to immunoprecipitation, the SDS was diluted to <0.1% with lysis buffer with protease inhibitors added. Samples were then subjected to immunoprecipitation with a rabbit polyclonal anti-Alexa Fluor 488 antibody (1μg/sample) overnight at 4°C and eluted off the protein A/G agarose with SDS-loading buffer. Alexa Fluor 488 was imaged using a Typhoon FLA 9500 and a rabbit monoclonal anti-LAMP1 antibody (Cell Signaling Technologies). Input was stained with Coomassie dye.

#### 1519 cross-linking to LAMP1 D1

1519 and competitor compounds in DMSO, or cholesterol and epicholesterol in acetone, were added to purified LAMP1 D1 in pH 5.5 lysis buffer to a final concentration of 10% DMSO or 10% DMSO and 2% acetone and incubated for 1h at 37°C. Samples were then irradiated for 7min at 365nm followed by 3min at 302nm and immediately used for click chemistry as above after the addition of SDS to a final concentration of 1%. Following click chemistry, gel-loading buffer was added to each sample and samples were analyzed by immunoblot using mouse monoclonal anti-His or anti-Alexa Fluor 488 antibodies (Thermo Fisher).

#### Photoclick cholesterol cross-linking to LAMP1 D1

Photoclick cholesterol and epicholesterol dissolved in acetone and 3.3 and 102 dissolved in DMSO were added to purified LAMP1 D1 in pH 5.5 lysis buffer to a final concentration of 10% DMSO and 2% acetone and incubated for 1h at 37°C. Samples were irradiated for 7min at 365nm followed by 3min at 302nm and immediately used for click chemistry as above after the addition of SDS to a final concentration of 1%. Following click chemistry, SDS-loading buffer was added to each sample and samples were used for immunoblot with anti-His or anti-Alexa Fluor 488 antibodies.

All data using these protocols have been confirmed in independent experiments.

### Co-immunoprecipitation

#### LASV GP—LAMP1 FL

293T LAMP1 KO cells transfected with pCAGGS-LASV GP-3xHA-6xHis and 293T pX459 cells were treated with the indicated concentrations of 3.3 in media (1% DMSO). Cells were lysed in pH 5.5 lysis buffer and lysates were mixed as indicated. Lysates were subjected to immunoprecipitation using an anti-His antibody and immunoblotted with anti-His and anti-LAMP1 antibodies.

#### LASV GP1-IgG or LCMV GP1-IgG—LAMP1 FL-His

293T cells transfected with pCAGGS-LAMP1 FL-3xFLAG-6xHis were lysed in pH 5.5 lysis buffer. Purified LASV GP1-IgG or purified LCMV GP1-IgG was added to the cell lysates and subjected to immunoprecipitation using protein A/G agarose beads (Santa Cruz Biotechnology) followed by immunoblot with anti-His and goat anti-human IgG (Santa Cruz Biotechnology) antibodies.

#### LASV GP1-IgG—LAMP1 D1

Purified LAMP1 D1-3xFLAG-6xHis was incubated with the indicated concentrations of cholesterol or epicholesterol and 3.3 or 102 in a final concentration of 2% acetone or 2% acetone and 10% DMSO for 1h at 37°C, mixed with LASV GP1-IgG complexed to protein A/G agarose and allowed to rotate overnight at 4°C. Samples were eluted off the beads and subjected to immunoblot with anti-human IgG and anti-His antibodies.

All data using these protocols have been confirmed in independent experiments.

### Thermal shift

#### Cellular thermal shift

293T cells were treated with the indicated concentrations of 3.3 or inactive derivatives for 1h at 37°C in 1% DMSO media. Cells were harvested in pH 5.5 PBS, heated to the indicated temperatures for 3min followed by incubation at 25°C for 3min. Lysis buffer was then added to the samples for a final concentration of 0.5% NP40 and cells were lysed by three cycles of freeze-thaw followed by centrifugation at 4°C for 20min at 14,000RPM to pellet cell debris and protein aggregates. Supernatants were collected and used for an immunoblot with anti-LAMP1 and rabbit monoclonal anti-LAMP2 antibodies (Abcam).

#### LAMP1 D1 thermal shift

LAMP1 D1 was incubated in pH 5.5 PBS with the indicated concentrations of 3.3 (1% DMSO final) or the indicated concentrations of cholesterol and epicholesterol (2% acetone final) for 30min at 37°C. Samples were then heated to the indicated temperatures for 3min followed by incubation at 25°C for 3min and centrifuged at 4°C for 20min at 14,000RPM. Supernatants were collected and used for an immunoblot with an anti-His antibody.

All data using these protocols have been confirmed in independent experiments.

### Compound docking and structure generation

The structure of LAMP1 D1 was obtained by threading the sequence of human LAMP1 D1 onto the structure of mouse LAMP2 D1 (PDB: 5GV3) using PHYRE^2^ (http://www.sbg.bio.ic.ac.uk/phyre2/html/page.cgi?id=index) [34]. 3.3 was docked using SwissDock (http://www.swissdock.ch/) [35]. Molecular graphics and analyses were performed with the UCSF Chimera package. Chimera is developed by the Resource for Biocomputing, Visualization, and Informatics at the University of California, San Francisco (supported by NIGMS P41-GM103311) [36].

### Chemical synthesis of 3.3, 100, 102, 103 and 1519

Standard resolution mass spectra were obtained on an Agilent 1200 Series HPLC (4.6 x 100 mm, 5 μm Phenomenex C18 reverse-phase column) and a 6130 Series mass spectrometer system; all mass spectra were obtained using electrospray ionization (EI) in either positive or negative ion mode. Standard reverse-phase HPLC conditions were as follows: mobile phase A = 0.1% formic acid in water; mobile phase B = 0.1% formic acid in acetonitrile. ^1^H NMR spectra were recorded on a Varian Inova 600 MHz spectrometer with chemical shifts reported in parts per million (ppm) relative to an internal standard (trimethylsilane). Coupling constants (*J*) are reported in hertz (Hz). Solvents for synthesis were purchased as anhydrous grade and used without further purification. Reagents were purchased from commercial sources and used as received.

### *Scheme 1 (*S6 Fig*)*: Synthesis of azirine-adamantane-1-carboxylic acid (2)

Azirine **2** was prepared similarly as reported in the literature [19, 37]. To a sealed tube at 0°C, containing a stir bar and acid **1** (225mg, 1.16mmol, 1.0 equiv) was added 7N NH3 in methanol (MeOH) (5.0mL). The reaction mixture was stirred for 3 h. Hydroxylamine-O-sulfonic acid (183mg, 1.62mmol, 1.4 equiv) was dissolved in MeOH (2mL) and added to the reaction mixture drop wise. The reaction mixture was allowed to warm to room temperature and was stirred for overnight, after which the reaction was centrifuged and the clear MeOH solution was collected, and concentrated. The residue was dissolved in 20% MeOH/CH2Cl2, and passed through a short silica gel column (eluent: 20% MeOH/CH2Cl2), then concentrated under reduced pressure.

The residue was then dissolved in anhyd. methanol (2.5mL), then cooled to 0°C, and Hunig’s base (0.2mL) was added. To the reaction was then added iodine solution in MeOH (concentration equals to 100 mg/1mL MeOH) in small portions, until a dark brown color persisted in the solution for more than 30min. The reaction was then treated with solid Na2S2O3, then acidified to pH 3 using aq. 1N HCl, then concentrated under reduced pressure. To the residue was added 15mL of 20% MeOH/CH2Cl2. The suspension was sonicated, filtered through a short silica gel column, and the filtrate concentrated to afford 186 mg of crude azirine acid 2 as an off white solid, which was taken to the next step directly. Azirine 2 was not detectable in either LCMS positive or negative mode. However, its structure is confirmed by the synthesis of 1519 (included in the later part of this experimental section).

### *Scheme 2 (*S7 Fig*)*: Synthesis of [(adamantane-1-carbonyl)-amino]-acetic acid (6)

In a 25mL round bottomed flask equipped with a stirring bar, under nitrogen, was placed glycine **4** (468mg) in 6mL of anhyd. tetrahydrofuran (THF). To the reaction was added N,N-Diisopropylethylamine (iPr2Net) (1.94mL), followed by acid chloride **3** (555mg) in 4mL of anhyd. THF. The reaction was stirred for 4h, then concentrated under reduced pressure. To the oily residue was added with water (50mL), and the suspension was sonicated for 10min. The white color solid was filtered, washed with water (2 x 50mL) to afford crude tert-butyl ester **5**, which was taken to the next step directly.

In a microwave reaction vial equipped with a stirring bar was placed the tert-butyl ester **5**. To the tube was added 4N HCl/dioxane (5mL). The reaction was stirred at r.t. for overnight. The reaction was then concentrated under reduced pressure to afford acid **6** (643mg, 97% for two steps), as a white solid. MS m/z: 236.2 (M-1), calc’d for C13H19NO3: 237.14.

### *Scheme 3 (*S8 Fig*)*: Synthesis of 4-[(2-methoxycarbonyl-phenyl)-phenyl-methyl]-piperazine-1-carboxylic acid tert-butyl ester (11-a), 4-[(2-carboxy-phenyl)-phenyl-methyl]-piperazine-1-carboxylic acid tert-butyl ester (11-b), 4-[(4-methoxycarbonyl-phenyl)-phenyl-methyl]-piperazine-1-carboxylic acid tert-butyl ester (11-c), and 4-(1-m-tolyl-ethyl)-piperazine-1-carboxylic acid tert-butyl ester (11-d)

In a 25mL round bottomed flask was placed 2-formyl-benzoic acid methyl ester 7-a (220mg, 1.34mmol), piperazine-1-carboxylic acid tert-butyl ester 9 (249mg, 1.34mmol), 1H-benzotriazole 8 (167mg, 1.41mmol). To the flask was added with toluene (5mL) and ethanol (EtOH) (1mL). The flask was sonicated until a homogeneous solution is achieved. The solvent was then removed under reduced pressure to dryness. To the residue was added with 10mL of toluene, and the solvent was again removed to dryness under reduced pressure to afford 4-[benzotriazol-1-yl-(2-methoxycarbonyl-phenyl)-methyl]-piperazine-1-carboxylic acid tert-butyl ester 10-a, which was used without further purification.

In a 25mL round bottomed flask equipped with a rubber septum, and a magnetic stirring bar was placed 4-[benzotriazol-1-yl-(2-methoxycarbonyl-phenyl)-methyl]-piperazine-1-carboxylic acid tert-butyl ester **10-a** (1.0mmol). Under the protection of nitrogen, 20mL of anhyd. THF was added. The reaction was then cooled to 0°C, and PhMgBr (1M in THF, 2.68mmol) was added drop wise. After 30min at 0°C, the reaction was warmed slowly to r.t., and stirred for overnight.

The reaction was then quenched with 2mL of MeOH, diluted with water (20mL), and extracted with ethyl acetate (EtOAc) (2 x 30mL). The combined organic phases was washed with brine (1 x 15mL), dried, and concentration under reduced pressure. The crude mixture was purified by TLC (3% MeOH/CH2Cl2) to afford two products: 1). 93mg (17%) of 4-[(2-methoxycarbonyl-phenyl)-phenyl-methyl]-piperazine-1-carboxylic acid tert-butyl ester **11-a**. MS m/z: 411.2(M+1), calc’d for C24H30N2O4: 410.22. 2). and the spontaneous hydrolysis product of the methyl ester **11-a**, which is the 4-[(2-carboxy-phenyl)-phenyl-methyl]-piperazine-1-carboxylic acid tert-butyl ester **11-b** (170 mg, 32%). MS m/z: 397.2.2(M+1), calc’d for C23H28N2O4: 396.20.

4-[(4-methoxycarbonyl-phenyl)-phenyl-methyl]-piperazine-1-carboxylic acid tert-butyl ester (**11c**), yield 43%, was prepared similarly as **11-a**. MS m/z: 411.2(M+1), calc’d for C24H30N2O4: 410.22.

4-(1-m-tolyl-ethyl)-piperazine-1-carboxylic acid tert-butyl ester (**11d**), yield 12%, was prepared similarly as **11**-a. MS m/z: 305.2(M+1), calc’d for C18H28N2O2: 304.22.

### *Scheme 4 (*S9 Fig*)*: Synthesis of 2-[(4-{2-[(adamantane-1-carbonyl)-amino]-acetyl}-piperazin-1-yl)-phenyl-methyl]-benzoic acid methyl ester (103), 4-[(4-{2-[(adamantane-1-carbonyl)-amino]-acetyl}-piperazin-1-yl)-phenyl-methyl]-benzoic acid (102), adamantane-1-carboxylic acid {2-oxo-2-[4-(1-m-tolyl-ethyl)-piperazin-1-yl]-ethyl}-amide (100), and adamantane-1-carboxylic acid [2-(4-benzhydryl-piperazin-1-yl)-2-oxo-ethyl]-amide (3.3)

The HCl/dioxane promoted de-Boc of **11-a**, **11-c**, **11-d**, to afford piperizines **12a**, **12-c**, **12-d** were carried out similarly as described for the synthesis of compound **6**. Piperizine **12-e** is purchased from Sigma-Aldrich.

In a sealed vial under nitrogen was placed acid **6** (9.24mg), N-(3-Dimethylaminopropyl)-N-ethylcarbodiimide (EDCI) (6mg), 1-Hydroxy-7-azabenzotriazole (HOAt) (6.9mg) in 2mL of anhydrous dichloromethane (DCM). iPr2NEt (15.5mg in 0.5mL of anhydrous DCM) was added, and the reaction was stirred for 1 hr. The activated acid was then added to a clear solution of piperazine **12-a** (10.4mg) and iPr2NEt (15.5mg) in 1mL of anhydr DCM. The reaction was then stirred for overnight. To the reaction was then added with 10mL of aq. 1N NaOH. The reaction was then extracted with CH2Cl2 (2 x 20mL). The combined organic phases was washed with water (10mL), dried with MgSO4, filtered, and concentrated under reduced pressure. The crude mixture was purified by TLC (3% MeOH/CH2Cl2) to afford 10.8mg (68%) of 2-[(4-{2-[(adamantane-1-carbonyl)-amino]-acetyl}-piperazin-1-yl)-phenyl-methyl]-benzoic acid methyl ester (**103**) as an oily wax. MS m/z: 530.2(M+1), calc’d for C32H39N3O4: 529.29. ^1^H NMR (400 MHz, CDCl_3_, ppm): δ 7.88 (d, 1H, J = 8.0), 7.71 (d, 1H, J = 8.0), 7.48–7.26 (m, 7 H), 6.78 (s, 1H), 5.34 (s, 1H), 3.97 (d, 2H, J = 2.8), 3.88 (s, 3H), 3.63–3.61 (m, 2H), 3.39–3.36 (m, 2H), 2.41–2.38 (m, 4H), 2.03–2.01 (m, 3H), 1.89–1.86 (m, 6H), 1.73–1.70 (m, 4H).

Adamantane-1-carboxylic acid {2-oxo-2-[4-(1-m-tolyl-ethyl)-piperazin-1-yl]-ethyl}-amide (**100**), yield 70%, was prepared similarly as **103**. m/z: 424.2(M+1), calc’d for C26H37N3O2: 423.29. ^1^H NMR (400 MHz, CDCl_3_, ppm): δ 7.25–7.08 (m, 4 H), 6.78 (s, 1H), 3.98 (d, 2H, J = 4.0), 3.63–3.60 (m, 2H), 3.39–3.35 (m, 3H), 2.47–2.38 (m, 4H), 2.03–2.01 (m, 3H), 1.89–1.85 (m, 6H), 1.71–1.69 (m, 4H), 1.36 (d, 3H, J = 6.4).

Adamantane-1-carboxylic acid [2-(4-benzhydryl-piperazin-1-yl)-2-oxo-ethyl]-amide (**3.3**) yield 82%, was prepared similarly as **103**. m/z: 472.2(M+1), calc’d for C30H37N3O2: 471.29. ^1^H NMR (400 MHz, CDCl_3_, ppm): δ 7.41–7.19 (m, 10 H), 6.77 (s, 1H), 4.25 (s, 1H), 3.98 (d, 2H, J = 1.6), 3.65–3.62 (m, 2H), 3.41–3.39 (m, 2H), 2.41–2.38 (m, 4H), 2.04–2.02 (m, 3H), 1.88–1.86 (m, 6H), 1.72–1.70 (m, 4H).

Methyl ester (**13**), yield 73%, was prepared similarly as **103**. m/z: 530.2(M+1), calc’d for C32H39N3O4: 529.29.

In a sealed vial was placed methyl ester **13** (53mg) in 5mL of THF and 1mL of water. To this reaction was added 0.5mL of LiOH solution (0.5M in water). The reaction was stirred vigorously for 6h at r.t. The reaction was then diluted with 10mL of water, extracted with EtOAc (1 x 10mL). To the aqueous layer, under stirring was added carefully 0.5mL of aq. HCl (0.5M in water). The aqueous layer was then extracted with CH2Cl2c (2 x 20mL). The combined organic phases was washed with water (10mL), dried with MgSO4, filtered, and concentrated under reduced pressure. The crude mixture was purified by TLC (5% MeOH/CH2Cl2) to afford 28mg (55%) of 4-[(4-{2-[(Adamantane-1-carbonyl)-amino]-acetyl}-piperazin-1-yl)-phenyl-methyl]-benzoic acid (**102**), m/z: 516.3(M+1), calc’d for C31H37N3O4: 515.28. ^1^H NMR (400 MHz, CDCl_3_, ppm): δ 8.02 (d, 1H, J = 8.0), 7.53 (d, 1H, J = 8.0), 7.39–7.21 (m, 7 H), 6.81 (s, 1H), 4.34 (s, 1H), 4.01 (d, 2H, J = 4.0), 3.64–3.60 (m, 2H), 3.38–3.34 (m, 2H), 2.41–2.37 (m, 4H), 2.03–2.00 (m, 3H), 1.89–1.83 (m, 6H), 1.74–1.70 (m, 4H).

### *Scheme 5 (*S10 Fig*)*: Synthesis of azirine-2-[(4-{2-[(adamantane-1-carbonyl)-amino]-acetyl}-piperazin-1-yl)-phenyl-methyl]-benzoic acid but-3-ynyl ester (1519)

In a sealed vial was placed acid **11-b** (57mg), K2CO3 (60mg) in 2mL of anhydr dimethylformamide (DMF). 4-Bromo-1-butyne **14** (38mg) in 0.5mL of anhyd. DMF was added slowly, and the reaction was stirred at r.t. for overnight. To the reaction was added water (10mL). The reaction was then extracted with CH2Cl2 (2 x 20mL). The combined organic phases was washed with water (10mL), dried with MgSO4, filtered, and concentrated under reduced pressure. The crude mixture was purified by TLC (3% MeOH/CH2Cl2) to afford 47mg (72%) of piperazine **15**. m/z: 449.2(M+1), calc’d for C27H32N2O4: 448.24.

The HCl/dioxane promoted de-Boc of **15**, to afford piperizine **16** was carried out similarly as described for the synthesis of compound **6**. m/z: 349.2(M+1), calc’d for C22H24N2O2: 348.18.

In a sealed vial was placed amine **16** (38mg) in anhyd THF (2mL). Glycine-ester **17** (33 mg) in 1mL of anhyd THF was added at r.t., followed by iPr2NEt (51mg). Reaction was stirred for overnight. The reaction was then quenched by addition of 2mL of aq. 1N NaOH. To the reaction was added water (10mL). The reaction was then extracted with CH2Cl2 (2 x 20mL). The combined organic phases was washed with water (10mL), dried with MgSO4, filtered, and concentrated under reduced pressure. The crude mixture was purified by TLC (3% MeOH/CH2Cl2) to afford 35mg (71%) of **18**. m/z: 506.2(M+1), calc’d for C29H35N3O5: 505.26.

The HCl/dioxane promoted de-Boc of **18**, to afford glycine-piperizines **19** was carried out similarly as described for the synthesis of compound **6**. m/z: 406.2(M+1), calc’d for C24H27N3O3: 405.21.

In a sealed vial, wrapped with aluminum foil, under nitrogen, was placed acid **2** (16mg), 1-[Bis(dimethylamino)methylene]-1H-1,2,3-triazolo[4,5-b]pyridinium 3-oxide hexafluorophosphate (HATU) (33mg) in 1mL of anhydrous DMF. iPr2NEt (26mg in 0.5mL of anhydrous DMF) was added, and the reaction was stirred for 0.5h. The activated acid was then added to a solution of piperazine **19** (22mg) and iPr2NEt (26mg) in 1mL of anhyd. DMF. The reaction was then stirred for overnight. To the reaction was then added with 10mL of water. The reaction was then extracted with CH2Cl2 (2 x 20mL). The combined organic phases was washed with water (10mL), dried with MgSO4, filtered, and concentrated under reduced pressure. The crude mixture was purified by TLC (3% MeOH/CH2Cl2) to afford 9.8mg (33%) of **1519** as a faint yellow solid. m/z: 594.3(M+1), calc’d for C35H39N5O4: 593.30. ^1^H NMR (400 MHz, CDCl_3_, ppm): δ 7.89 (d, 1H, J = 8.0), 7.72 (d, 1H, J = 8.0), 7.49–7.25 (m, 7 H), 6.84 (s, 1H), 5.39 (s, 1H), 4.41 (t, 2H, J = 6.4), 3.99 (d, 2H, J = 3.6), 3.63–3.61 (m, 2H), 3.43–3.39 (m, 2H), 2.65 (t, 2H, J = 6.4), 2.43–2.40 (m, 4H), 2.04–2.02 (m, 4H), 1.88–1.86 (m, 4H), 1.72–1.70 (m, 4H).

## Supporting information

S1 Fig Structure and activity of 3.3-related compounds on transduction by LASV GP-pseudotyped MLV and infection by rLASV-eGFP. (A) Structures of 3.3-related compounds 3.0, 100, 102 and 103. (B) Effect of 3.3 analogs 3.0, 100, 102 and 103 on transduction of Vero cells by MLV encoding GFP and pseudotyped with LASV GP. Target cells were incubated with the indicated compounds for 1h before virus challenge. IC_50_ is the concentration (μM) of compound required to reduce infection by 50% (n = 2 or 3). (C) 3.3 inhibits infection by rLASV-eGFP. Vero cells were incubated with 3.3 at the indicated concentrations for 1h before infection with a recombinant LASV expressing eGFP (rLASV-eGFP). IC_50_ is the concentration (μM) of 3.3 required to reduce infection by 50% after 48 hours (n = 2). The cytotoxicity was measured after treatment of cells with 3.3 at the indicated concentrations for 48h.
(TIF)

S2 Fig CRISPR-mediated knockout (KO) of LAMP1 in 293T and Huh7.5 cells. (A) Cells from the indicated cell lines were lysed and subjected to immunoblot with anti-tubulin and anti-LAMP1 antibodies. (B) gDNA isolated from the indicated cell lines was sequenced around the CRISPR cut site.
(TIF)

S3 Fig LASV GP1-IgG can bind to LAMP1 from cells. Purified LASV GP1-IgG or purified LCMV GP1-IgG was added to lysates from cells expressing LAMP1-His. Samples were subjected to immunoprecipitation against human IgG and bound LAMP1 was detected with an anti-His antibody.
(TIF)

S4 Fig Cholesterol and 3.3 binding protect LAMP1 D1 from thermal denaturation. (A) Thermal denaturation profile of purified LAMP1 D1. LAMP1 D1-His was heated to the indicated temperatures for 3min. Samples were centrifuged and supernatants were analyzed by immunoblot with an anti-His antibody. (B) Cholesterol (top) but not epicholesterol (bottom) dose-dependently protects purified LAMP1 D1 from thermal denaturation at 80°C. Purified LAMP1 D1-His was incubated with the indicated concentrations of cholesterol or epicholesterol for 30min at 37°C prior to being heated to the indicated temperatures. Samples were centrifuged and the supernatants were analyzed by immunoblot with an anti-His antibody. (C) 3.3 dose-dependently protects purified LAMP1 D1 from thermal denaturation at 80°C. Purified LAMP1 D1-His was incubated with the indicated concentrations of 3.3 for 30min at 37°C prior to being heated to 80°C. Samples were centrifuged and the supernatants were analyzed by immunoblot with an anti-His antibody.
(TIF)

S5 Fig Predicted LAMP1 D1 surface view. (A) Predicted LAMP1 D1 surface view colored by Kyte-Doolittle hydrophobicity. Orange: most hydrophobic. Blue: least hydrophobic. Inset shows a close-up view of the hydrophobic pocket. (B) 3.3 (yellow) docked onto the predicted LAMP1 D1 structure. The adamantane group is predicted to be buried in the hydrophobic pocket while the diphenyl moiety makes contacts with hydrophobic residues outside of the pocket on the surface of LAMP1 D1. Arrows label the locations of residues I142 and V161 predicted to contact 3.3.
(TIF)

S6 Fig Synthesis of azirine-adamantane-1-carboxylic acid (2). (TIF)

S7 Fig Synthesis of [(adamantane-1-carbonyl)-amino]-acetic acid (6). (TIF)

S8 Fig Synthesis of 4-[(2-methoxycarbonyl-phenyl)-phenyl-methyl]-piperazine-1-carboxylic acid tert-butyl ester (11-a), 4-[(2-carboxy-phenyl)-phenyl-methyl]-piperazine-1-carboxylic acid tert-butyl ester (11-b), 4-[(4-methoxycarbonyl-phenyl)-phenyl-methyl]-piperazine-1-carboxylic acid tert-butyl ester (11-c), and 4-(1-m-tolyl-ethyl)-piperazine-1-carboxylic acid tert-butyl ester (11-d). (TIF)

S9 Fig Synthesis of 2-[(4-{2-[(adamantane-1-carbonyl)-amino]-acetyl}-piperazin-1-yl)-phenyl-methyl]-benzoic acid methyl ester (103), 4-[(4-{2-[(adamantane-1-carbonyl)-amino]-acetyl}-piperazin-1-yl)-phenyl-methyl]-benzoic acid (102), adamantane-1-carboxylic acid {2-oxo-2-[4-(1-m-tolyl-ethyl)-piperazin-1-yl]-ethyl}-amide (100), and adamantane-1-carboxylic acid [2-(4-benzhydryl-piperazin-1-yl)-2-oxo-ethyl]-amide (3.3). (TIF)

S10 Fig Synthesis of azirine-2-[(4-{2-[(adamantane-1-carbonyl)-amino]-acetyl}-piperazin-1-yl)-phenyl-methyl]-benzoic acid but-3-ynyl ester (1519). (TIF)
