# Supplementary figures and images for: Critical role for cholesterol in Lassa fever virus entry identified by a novel small molecule inhibitor targeting the viral receptor LAMP1
Source: PLoS Pathog. 2018 Sep 28;14(9):e1007322. doi: 10.1371/journal.ppat.1007322 (PMC6179309; doi:10.1371/journal.ppat.1007322)

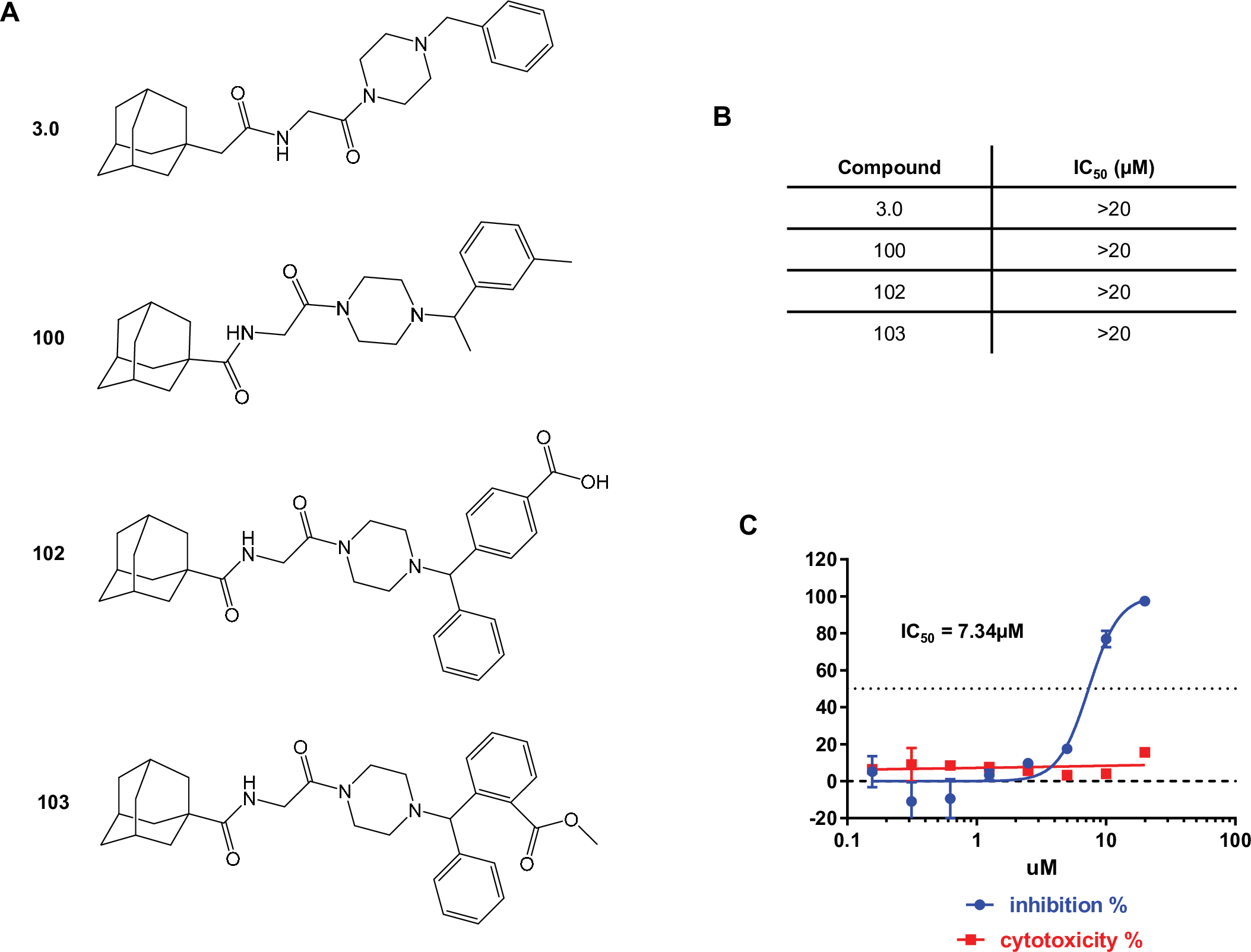

Supplement: S1 Fig — (A) Structures of 3.3-related compounds 3.0, 100, 102 and 103. (B) Effect of 3.3 analogs 3.0, 100, 102 and 103 on transduction of Vero cells by MLV encoding GFP and pseudotyped with LASV GP. Target cells were incubated with the indicated compounds for 1h before virus challenge. IC50 is the concentration (μM) of compound required to reduce infection by 50% (n = 2 or 3). (C) 3.3 inhibits infection by rLASV-eGFP. Vero cells were incubated with 3.3 at the indicated concentrations for 1h before infection with a recombinant LASV expressing eGFP (rLASV-eGFP). IC50 is the concentration (μM) of 3.3 required to reduce infection by 50% after 48 hours (n = 2). The cytotoxicity was measured after treatment of cells with 3.3 at the indicated concentrations for 48h. (TIF) [file ppat.1007322.s001.tif]

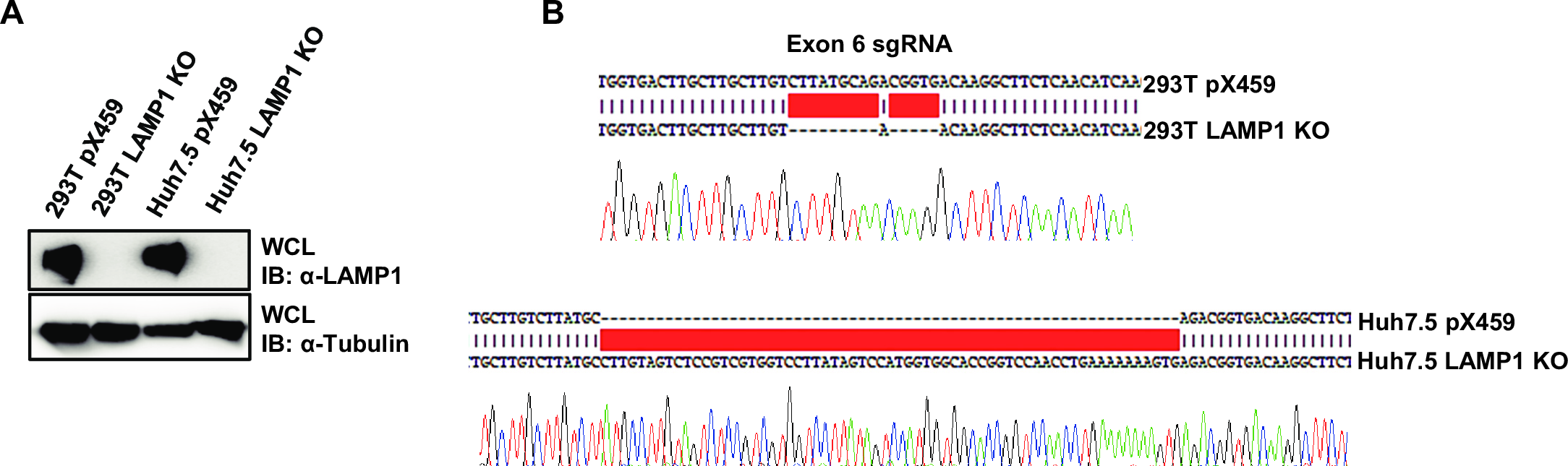

Supplement: S2 Fig — (A) Cells from the indicated cell lines were lysed and subjected to immunoblot with anti-tubulin and anti-LAMP1 antibodies. (B) gDNA isolated from the indicated cell lines was sequenced around the CRISPR cut site. (TIF) [file ppat.1007322.s002.tif]

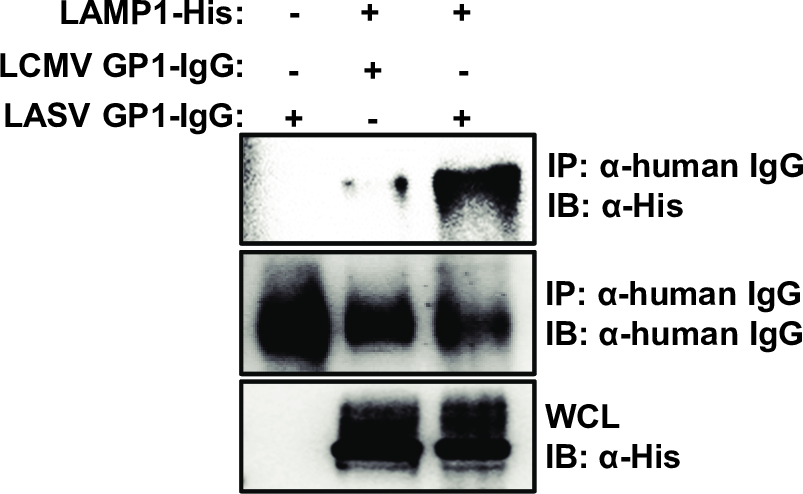

Supplement: S3 Fig — Purified LASV GP1-IgG or purified LCMV GP1-IgG was added to lysates from cells expressing LAMP1-His. Samples were subjected to immunoprecipitation against human IgG and bound LAMP1 was detected with an anti-His antibody. (TIF) [file ppat.1007322.s003.tif]

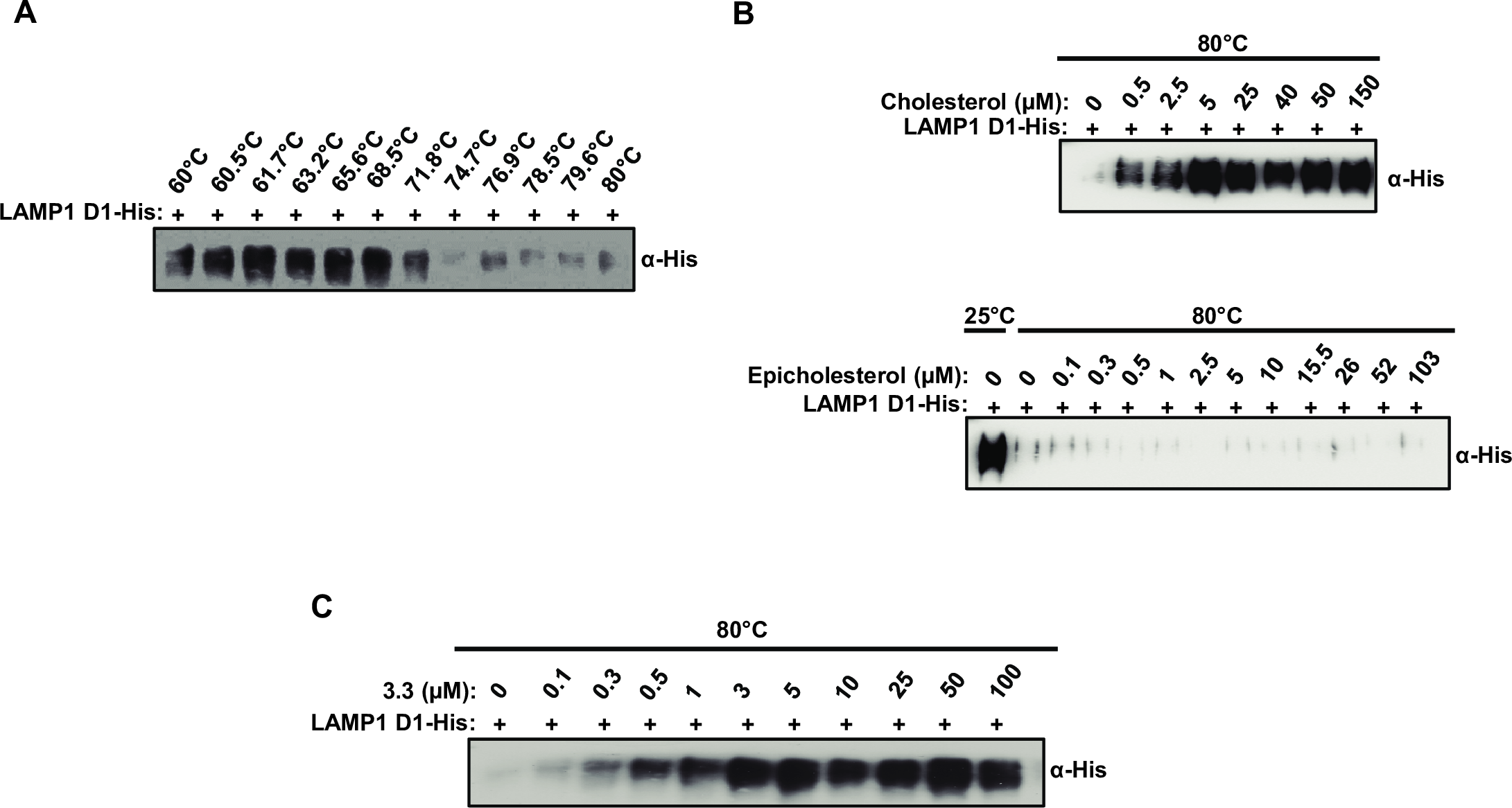

Supplement: S4 Fig — (A) Thermal denaturation profile of purified LAMP1 D1. LAMP1 D1-His was heated to the indicated temperatures for 3min. Samples were centrifuged and supernatants were analyzed by immunoblot with an anti-His antibody. (B) Cholesterol (top) but not epicholesterol (bottom) dose-dependently protects purified LAMP1 D1 from thermal denaturation at 80°C. Purified LAMP1 D1-His was incubated with the indicated concentrations of cholesterol or epicholesterol for 30min at 37°C prior to being heated to the indicated temperatures. Samples were centrifuged and the supernatants were analyzed by immunoblot with an anti-His antibody. (C) 3.3 dose-dependently protects purified LAMP1 D1 from thermal denaturation at 80°C. Purified LAMP1 D1-His was incubated with the indicated concentrations of 3.3 for 30min at 37°C prior to being heated to 80°C. Samples were centrifuged and the supernatants were analyzed by immunoblot with an anti-His antibody. (TIF) [file ppat.1007322.s004.tif]

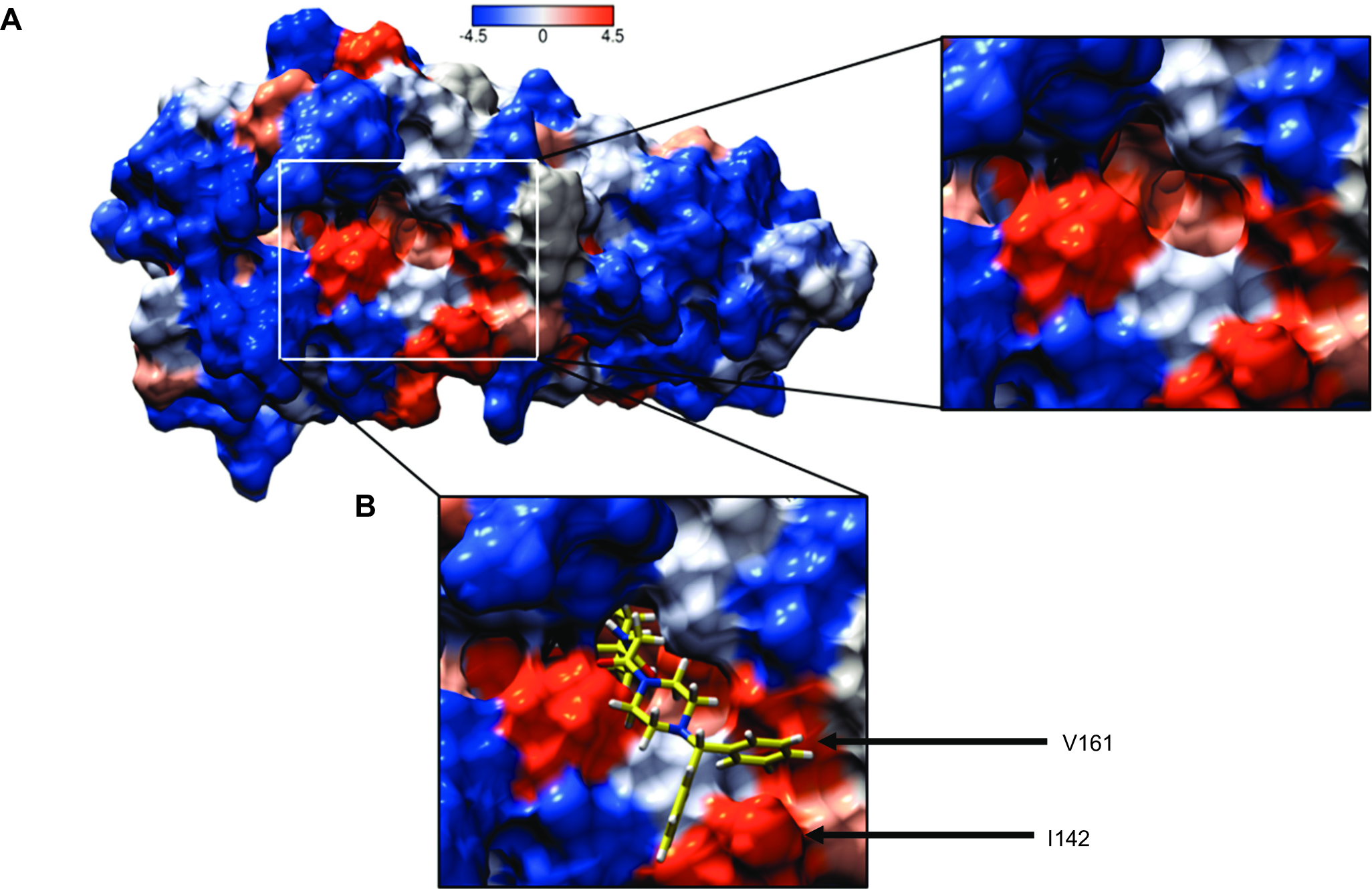

Supplement: S5 Fig — (A) Predicted LAMP1 D1 surface view colored by Kyte-Doolittle hydrophobicity. Orange: most hydrophobic. Blue: least hydrophobic. Inset shows a close-up view of the hydrophobic pocket. (B) 3.3 (yellow) docked onto the predicted LAMP1 D1 structure. The adamantane group is predicted to be buried in the hydrophobic pocket while the diphenyl moiety makes contacts with hydrophobic residues outside of the pocket on the surface of LAMP1 D1. Arrows label the locations of residues I142 and V161 predicted to contact 3.3. (TIF) [file ppat.1007322.s005.tif]

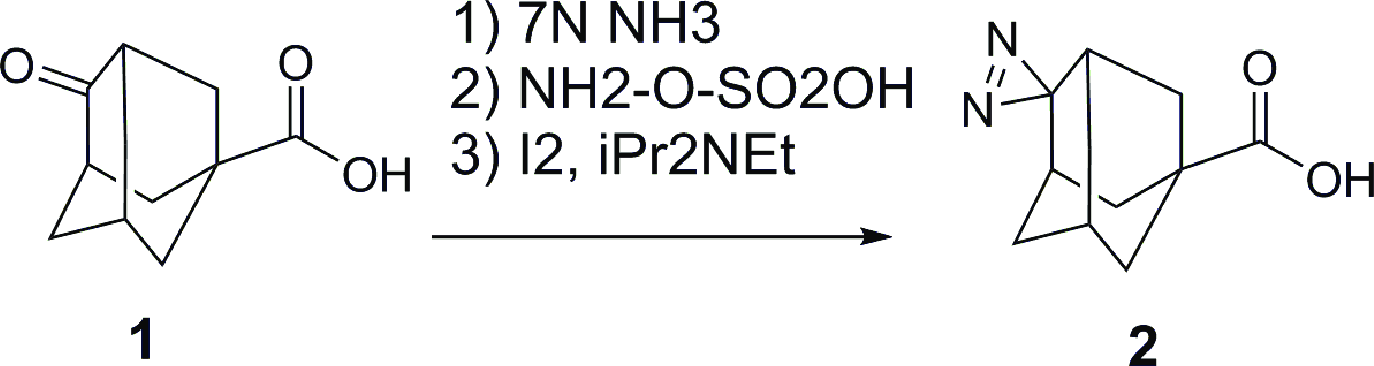

Supplement: S6 Fig — (TIF) [file ppat.1007322.s006.tif]

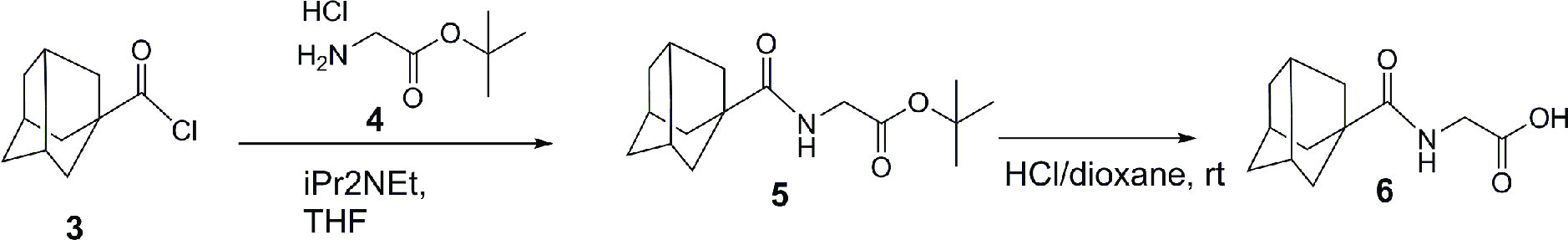

Supplement: S7 Fig — (TIF) [file ppat.1007322.s007.tif]

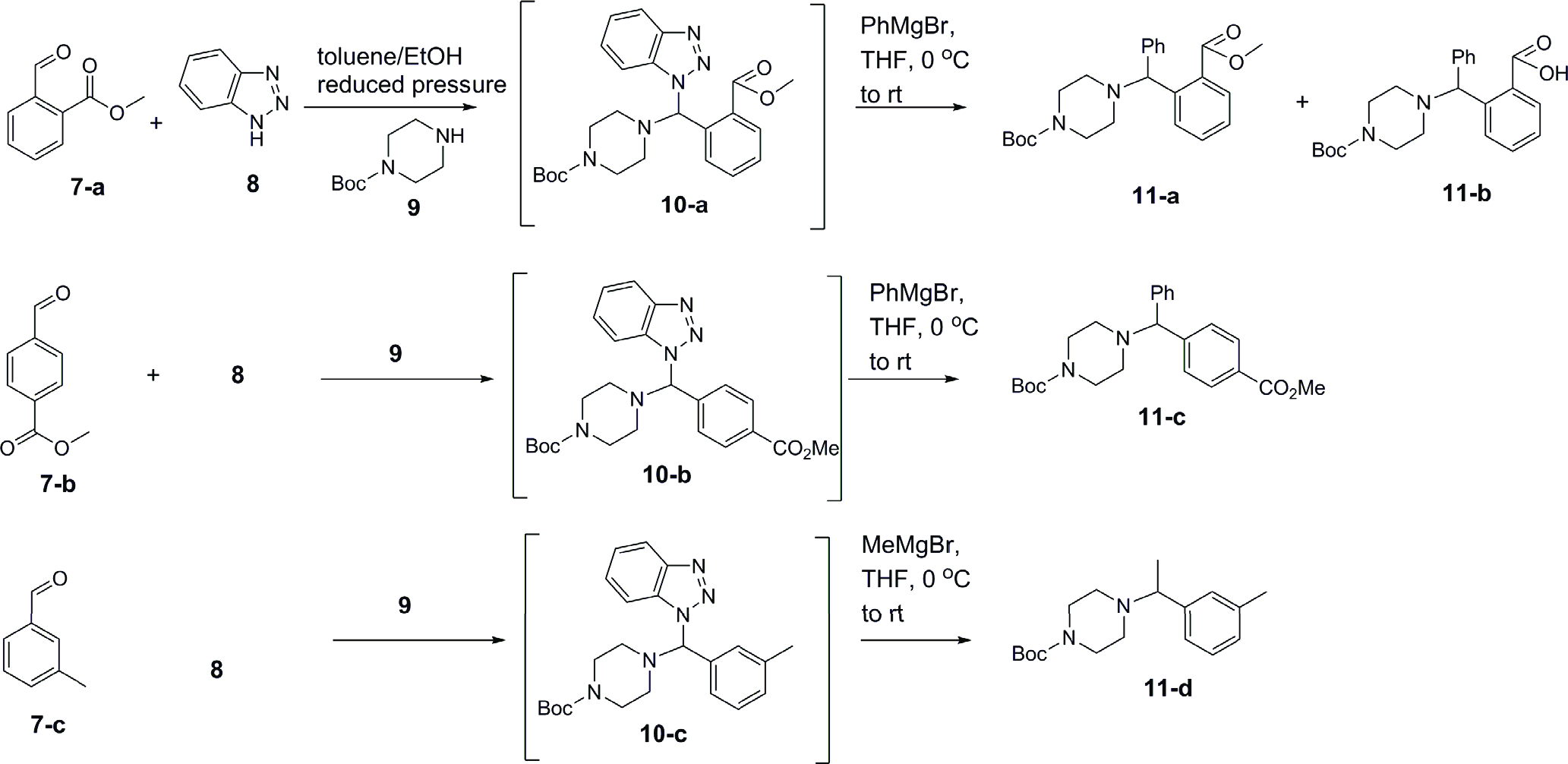

Supplement: S8 Fig — (TIF) [file ppat.1007322.s008.tif]

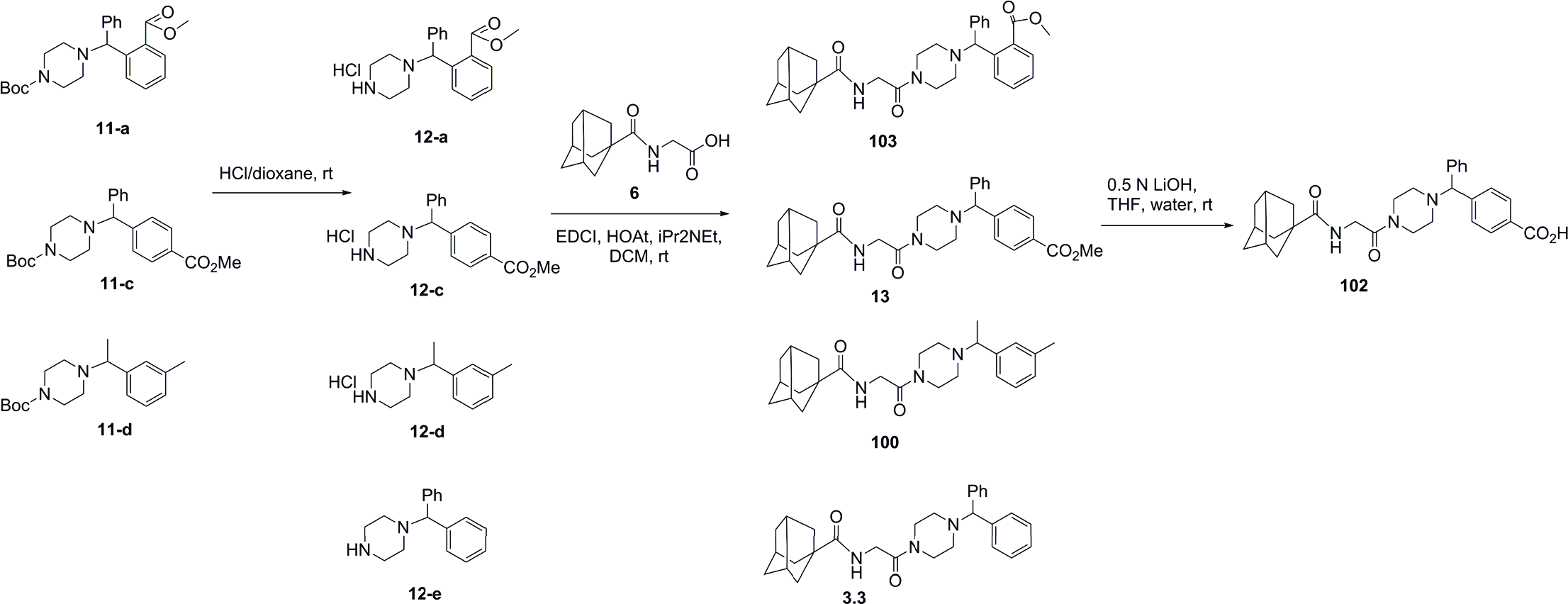

Supplement: S9 Fig — (TIF) [file ppat.1007322.s009.tif]

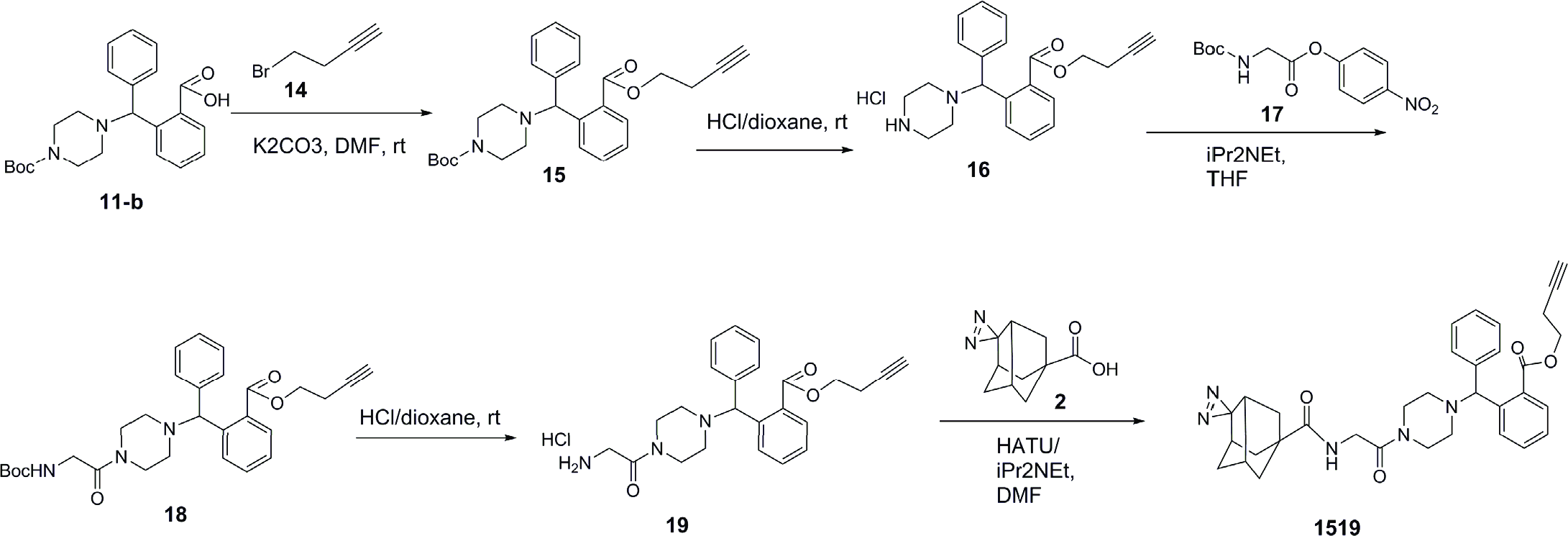

Supplement: S10 Fig — (TIF) [file ppat.1007322.s010.tif]
